# PRMT1‐Mediated SWI/SNF Complex Recruitment via SMARCC1 Drives IGF2BP2 Transcription to Enhance Carboplatin Resistance in Head and Neck Squamous Cell Carcinoma

**DOI:** 10.1002/advs.202417460

**Published:** 2025-04-24

**Authors:** Shixian Liu, Wentao Zhang, Weiwei Liu, Zhao Ding, Ruijing Zhang, Yuefeng Han, Zihao Niu, Mengdie Zhang, Hui Li, Dapeng Li, Zixi Wang, Jie Peng, Yu Wu, Yanxun Han, Zihui Xie, Jing Wu, Liang Qin, Zhongdong Hu, Xu Chen, Yunlong Hu, Yehai Liu, Shiyin Ma, Xiaojun Zha

**Affiliations:** ^1^ Department of Otolaryngology Head & Neck Surgery The First Affiliated Hospital of Anhui Medical University Hefei 230022 China; ^2^ Department of Otolaryngology Head & Neck Surgery The First Affiliated Hospital of Bengbu Medical University Bengbu 233004 China; ^3^ Department of Biochemistry & Molecular Biology School of Basic Medicine Anhui Medical University Hefei 230032 China; ^4^ Department of Otolaryngology Head and Neck Surgery and Scientific Research and Experiment Center The Affiliated Bozhou Hospital of Anhui Medical University Bozhou 236800 China; ^5^ Institutes of Biomedical Sciences Children's Hospital of Fudan University National Children's Medical Center Fudan University Shanghai 200032 China; ^6^ Department of Urology Shanghai General Hospital Shanghai Jiao Tong University School of Medicine Shanghai 200080 China; ^7^ Modern Research Center for Traditional Chinese Medicine Beijing Research Institute of Chinese Medicine Beijing University of Chinese Medicine Beijing 100029 China; ^8^ Department of Otolaryngology Head and Neck Surgery Anhui NO.2 Provincial People's Hospital Hefei 230041 China

**Keywords:** chemoresistance, HNSCC, IGF2BP2, PRMT1, SWI/SNF Complex

## Abstract

Head and neck squamous cell carcinoma (HNSCC) is a malignancy with poor prognosis and chemotherapy resistance. Here, protein arginine methyltransferase 1 (PRMT1) is identified as a key driver of carboplatin (CBP) resistance in HNSCC. Analyses of clinical samples, cell lines, patient‐derived organoids, and xenograft models reveal that PRMT1 promotes tumor growth and CBP resistance through a novel, methyltransferase‐independent mechanism. Conditional PRMT1 knockout suppresses tumorigenesis and enhances CBP sensitivity in vivo, highlighting its essential role in HNSCC progression. Mechanistically, PRMT1 recruits the SWI/SNF chromatin remodeling complex via direct interaction with SMARCC1, leading to the transcriptional activation of insulin‐like growth factor 2 mRNA‐binding protein 2 (IGF2BP2), which enhances CBP resistance and tumor growth. Notably, this function is independent of PRMT1's enzymatic activity, distinguishing it from its well‐established roles in arginine methylation. Furthermore, pre‐B‐cell leukemia homeobox 2 (PBX2) is identified as an upstream transcriptional activator that binds the PRMT1 promoter, driving its overexpression and reinforcing this oncogenic network. Clinically, high PBX2, PRMT1, SMARCC1, and IGF2BP2 expression correlates with malignant progression and poor prognosis in HNSCC patients. This study uncovers a previously unrecognized non‐catalytic function of PRMT1 and highlights the PBX2‐PRMT1‐SWI/SNF‐IGF2BP2 axis as a potential therapeutic target for overcoming CBP resistance in HNSCC.

## Graphical abstract

PRMT1 drives carboplatin resistance and tumor progression in head and neck squamous cell carcinoma (HNSCC) through a novel, methyltransferase‐independent mechanism. It recruits the SWI/SNF complex to activate IGF2BP2, promoting tumor growth and carboplatin resistance. PBX2 upregulates PRMT1, reinforcing this pathway. This study uncovers a non‐catalytic function of PRMT1, offering a potential therapeutic target for overcoming carboplatin resistance in HNSCC.

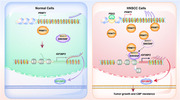

## 1 Introduction

Head and neck cancer is the sixth most common malignancy worldwide, with head and neck squamous cell carcinoma (HNSCC) accounting for over 90% of cases.^[^ ^1^ ^]^ HNSCC is characterized by high morbidity, mortality, and poor prognosis, with ≈600 000 new cases and a 40–50% mortality rate annually.^[^ ^2^ ^]^ These cancers typically arise from the hypopharynx, oropharynx, nasopharynx, and oral cavity and are mainly caused by smoking, alcohol consumption, and high‐risk HPV16 infection.^[^ ^3^ ^]^ Treatment for HNSCC includes surgery, chemotherapy, targeted therapy, and radiotherapy.^[^ ^4^ ^]^ While immune checkpoint inhibitors have revolutionized cancer therapy, their efficacy in HNSCC is limited by low response rates and severe immune‐related adverse events.^[^ ^5^ ^]^ Platinum‐based chemotherapy, primarily cisplatin and carboplatin (CBP), remains the cornerstone of treatment, particularly for advanced or inoperable cases.^[^ ^6^ ^]^ However, both inherent and acquired resistance to platinum therapies often leads to treatment failure, relapse, and even death.^[^ ^7^ ^]^ Therefore, understanding the mechanisms behind platinum resistance is critical for developing more effective treatments for HNSCC.

Protein arginine methylation, catalyzed by protein arginine methyltransferases (PRMTs), plays a key role in regulating cellular functions.^[^ ^8^ ^]^ Among the PRMT family, PRMT1 is the most abundant, responsible for ≈85% of cellular PRMT activity.^[^ ^9^ ^]^ It catalyzes both monomethylation and asymmetric dimethylation of arginine residues on target proteins.^[^ ^10^ ^]^ PRMT1 is essential for various biological processes, including transcription, RNA metabolism, and signal transduction.^[^ ^11^ ^]^ Elevated PRMT1 levels have been linked to several cancers, where they contribute to tumor initiation and progression.^[^ ^12^ ^]^ Recent studies suggest that PRMT1 also plays a significant role in chemoresistance. For instance, in triple‐negative breast cancer, PRMT1 promotes paclitaxel resistance by methylating enzymes like PKM2 and PHGDH.^[^ ^13^ ^]^ Similarly, PRMT1 has been implicated in acquired gemcitabine resistance in pancreatic cancer.^[^ ^14^ ^]^ Despite these findings, the role of PRMT1 in chemotherapy resistance in HNSCC remains poorly understood.

In this study, we identified PRMT1 as a key driver of CBP resistance in HNSCC cells. We validated this finding across multiple models, including cell line‐derived xenografts (CDX), patient‐derived organoids (PDO), patient‐derived xenografts (PDX), and PRMT1 conditional knockout (cKO) mouse models. We showed that PRMT1 recruits the SWI/SNF chromatin remodeling complex via its interaction with SWI/SNF related, matrix associated, actin dependent regulator of chromatin subfamily C member 1 (SMARCC1), promoting insulin‐like growth factor 2 mRNA‐binding protein 2 (IGF2BP2) transcription in an enzyme‐independent manner. This mechanism drives CBP resistance and tumor growth. Additionally, we found that the transcription factor PBX2 binds to the PRMT1 promoter, enhancing its expression and elevating IGF2BP2 levels in HNSCC cells. Clinical data analysis revealed a strong correlation between high levels of PBX2, PRMT1, SMARCC1, and IGF2BP2 with poor prognosis and tumor progression in HNSCC patients. Our findings establish the PBX2‐PRMT1‐SWI/SNF‐IGF2BP2 axis as a critical mechanism underlying CBP resistance in HNSCC.

## 2 Results

### 2.1 PRMT1 is Highly Expressed in HNSCC and Contributes to the Increased Resistance of HNSCC Cells to CBP

**Figure 1:**
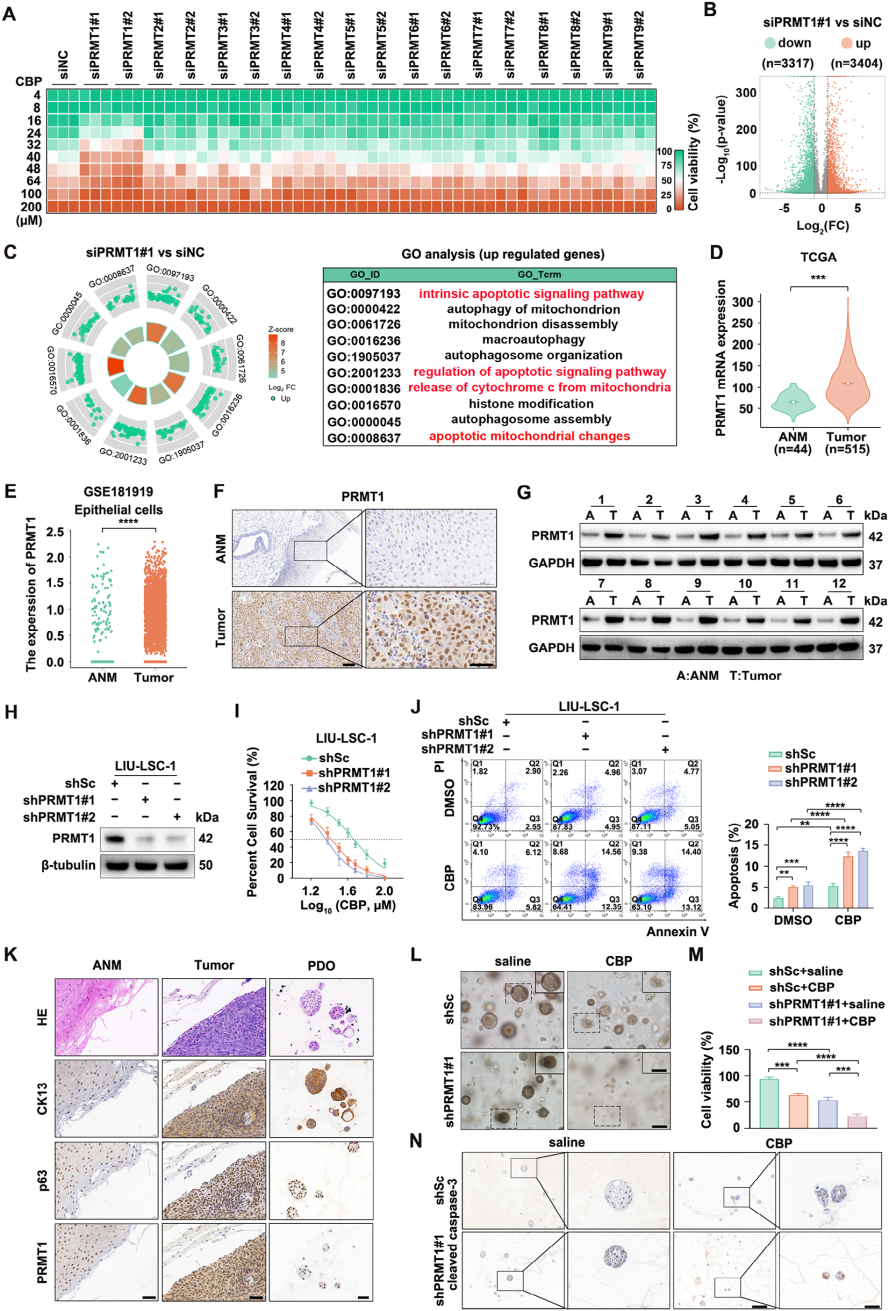
Overexpression of PRMT1 in HNSCC enhances resistance to CBP. A) The heatmap indicates that the knockdown of PRMT1, unlike other members of the PRMT family, enhances the susceptibility of FaDu cells to CBP. B, C) PRMT1 knockdown (siPRMT1#1) and control (siNC) FaDu cells were treated with CBP (45 µM) for 72 h, followed by RNA‐seq (RNA sequencing). A volcano plot of DEGs is shown in B), and GO BP analysis was performed to identify the top 10 biological processes associated with these DEGs C). D) PRMT1 expression in HNSCC tumor tissues (*n* = 515) and ANM tissues (*n* = 44) were analyzed using TCGA‐HNSCC data. Error bars represent mean ± SD, with ***p < 0.001. E) PRMT1 levels in 24 HNSCC tissues and 9 ANM samples were analyzed using data from GSE181919, with normalized PRMT1 expression levels in individual cells presented in a dot plot. The significance level is indicated as ****p < 0.0001. F,G) Paired ANM and tumor tissues (*n* = 12) were analyzed via IHC F) and western blotting G), with representative IHC images shown in panel G. Scale bars represent 100 µm (low magnification) and 50 µm (high magnification). H‐J) LIU‐LSC‐1 cells were transduced with lentiviruses expressing shRNAs targeting PRMT1 (shPRMT1#1 and shPRMT1#2) or a control scrambled sequence (shSc). Western blotting analysis was used to assess PRMT1 protein expression levels H). The IC50 values of CBP were determined using the CCK‐8 assay I). Additionally, cells treated with CBP (45 µM, 24 h) or DMSO were analyzed for apoptosis using flow cytometry J). Error bars indicate mean ± SD, **p < 0.01, ***p < 0.001, ****p < 0.0001. K) Representative IHC images of CK13, p63, and PRMT1 staining from paired ANM and HNSCC tissues, as well as organoids, are presented. Scale bar: 50 µm. L–N) HNSCC organoids infected with shPRMT1#1 or shSc lentiviruses were treated with CBP (45 µM) or saline for 48 h. Cell viability was assessed using the Cell‐Titer Glo‐3D assay. Panel L) shows representative phase contrast images, M) presents the quantified data, and N) displays representative IHC images of cleaved caspase‐3 staining. Scale bars represent 200 µm (low magnification) and 50 µm (high magnification). Error bars represent mean ± SD, **p < 0.01, ***p < 0.001 ****p < 0.0001.

To identify the most critical PRMT for CBP resistance in HNSCC, we used siRNA to knock down PRMT1–9 in FaDu cells and assessed their survival under varying CBP concentrations. Knockdown efficiency was confirmed by quantitative real‐time PCR (qRT‐PCR) and western blotting (Figure S1A–J, Supporting Information). Among the PRMT family, PRMT1 knockdown significantly increased CBP sensitivity, whereas PRMT8 showed minimal expression (**Figure** **1**A; Figure S1A,I, Supporting Information). RNA sequencing identified 6721 differentially expressed genes (DEGs) in CBP‐treated PRMT1‐knockdown cells, including 3404 upregulated and 3317 downregulated genes. Gene Ontology (GO) Biological Process (BP) analysis revealed that upregulated genes were enriched in apoptosis, autophagy, and histone modification pathways (Figure 1B,C; Table S1, Supporting Information), while downregulated genes were associated with small GTPase‐mediated signal transduction, Golgi vesicle transport, amino acid metabolism, etc. (Figure S2A, Supporting Information). Functional validation demonstrated that inhibiting apoptosis—but not autophagy—rescued CBP sensitivity induced by PRMT1 knockdown (Figure S2B,C, Supporting Information), suggesting PRMT1 confers CBP resistance primarily by suppressing apoptosis.

We then examined PRMT1 expression in HNSCC. Analysis of the TCGA dataset and single‐cell RNA‐seq data (GSE181919) revealed significant PRMT1 upregulation in HNSCC tissues compared to adjacent normal mucosa (ANM) (Figure 1D,E). immunohistochemical staining (IHC), western blotting, and qRT‐PCR analyses of 12 paired HNSCC and ANM samples confirmed this trend (Figure 1F,G; Figure S3A, Supporting Information). Similarly, PRMT1 mRNA and protein levels were elevated in HNSCC cell lines (Figure S3B, Supporting Information), with nuclear localization confirmed by Immunofluorescence (IF) analysis (Figure S3C, Supporting Information).

To select suitable cell lines for functional studies, we assessed CBP 50% inhibitory concentration (IC50) values across multiple HNSCC cell lines and found a positive correlation between CBP resistance and PRMT1 expression (Figure S3D, Supporting Information). LIU‐LSC‐1 cells (high PRMT1, CBP‐resistant) were chosen for PRMT1 knockdown, while TU177 cells (low PRMT1, CBP‐sensitive) were selected for overexpression. Successful modulation of PRMT1 was validated by western blotting and qRT‐PCR (Figure 1H; Figure S3E,F, Supporting Information). Cytotoxicity assays (Figure 1I; Figure S3G, Supporting Information), colony formation assays (Figure S3H,I, Supporting Information), and Annexin‐V/PI staining (Figure 1J; Figure S3J, Supporting Information) confirmed that PRMT1 silencing enhanced CBP sensitivity, while overexpression increased resistance. Moreover, restoring PRMT1 in knockdown cells with a shRNA‐resistant construct rescued CBP resistance (Figure S3K–M, Supporting Information).

Using PDO models, which preserve tumor heterogeneity and histological characteristics, we further evaluated the impact of PRMT1 silencing on CBP response. Histological analysis confirmed that HNSCC organoids retained original tumor features (Figure 1K). Consistently, PRMT1 depletion significantly enhanced CBP's inhibitory effect on organoid growth (Figure 1L–N; Figure S3N, Supporting Information). In summary, PRMT1 is highly expressed in HNSCC and plays a key role in CBP resistance by suppressing apoptosis, making it a potential therapeutic target.

### 2.2 Depletion of PRMT1 Potentiates the Efficacy of CBP In Vivo

The data above prompted us to explore whether PRMT1 depletion enhances CBP's anti‐tumor efficacy in vivo. We established a CDX model by subcutaneously inoculating shPRMT1#1 LIU‐LSC‐1 and control cells into nude mice. Tumor‐bearing animals received intraperitoneal CBP or saline for two weeks. As shown in Figure S4A–D (Supporting Information), PRMT1 knockdown significantly suppressed tumor growth and enhanced CBP's anti‐tumor effect. Notably, no significant weight loss was observed (Figure S4E, Supporting Information), suggesting minimal toxicity. IHC staining further revealed reduced Ki67 expression and increased cleaved caspase‐3 levels following CBP treatment, effects amplified by PRMT1 depletion (Figure S4F, Supporting Information).

**Figure 2:**
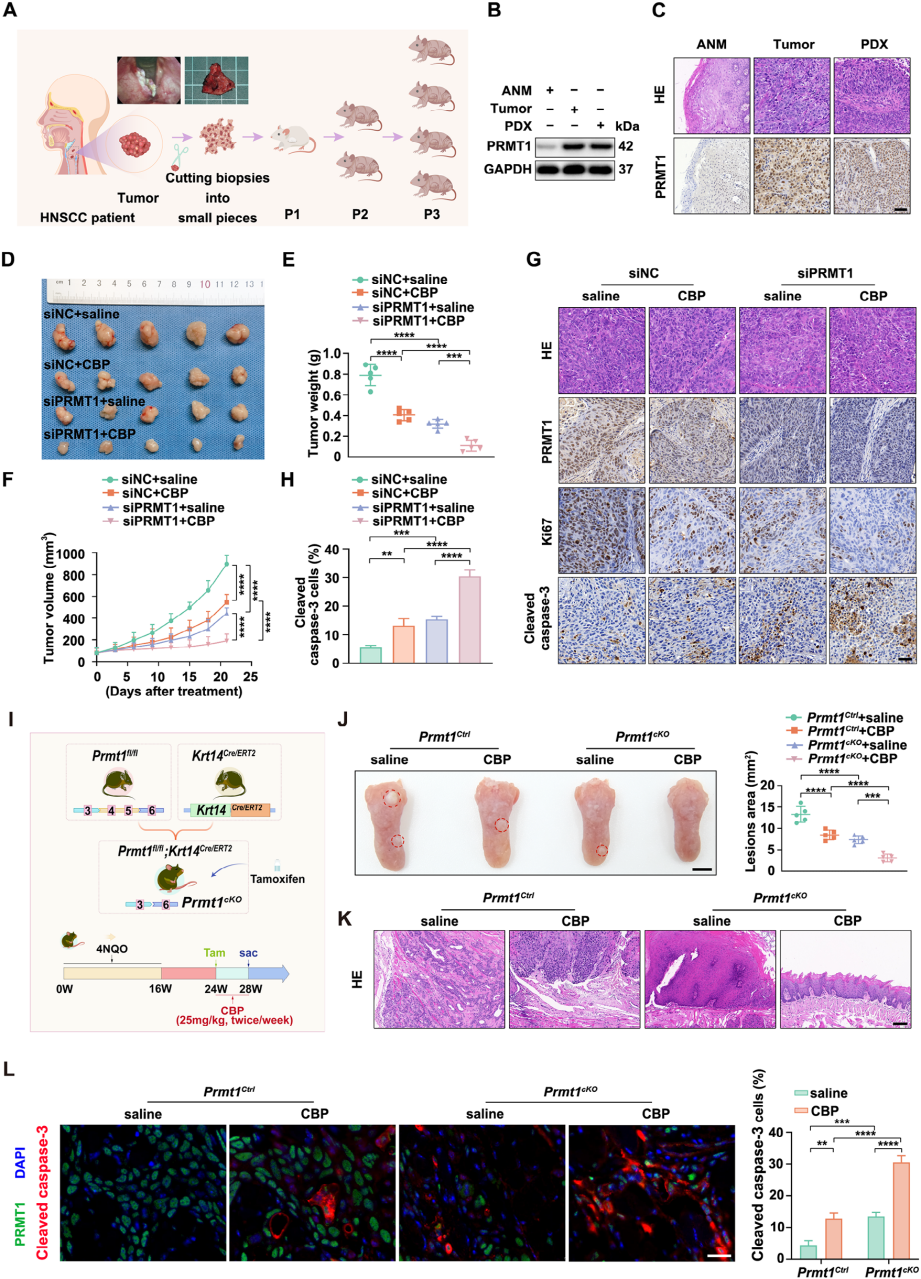
Depletion of PRMT1 boosts the effectiveness of CBP in vivo. A) Workflow diagram depicting the establishment of HNSCC PDX models. B,C) Western blotting (B) and IHC C) analysis of PRMT1 expression in PDX tumor tissues, primary HNSCC tissues, and paired ANM tissues. Scale bar: 100 µm. D–H) Evaluation of tumor characteristics, including tumor images D), tumor weight E), and tumor volume F) in PDX models after treatment with PRMT1 siRNAs or siNC combined with CBP (25 mg kg^−1^, i.p., twice/week) or saline (*n* = 5 mice per group). Representative IHC images showing PRMT1, Ki67, and cleaved caspase‐3 staining in PDX tumors G). Scale bar: 50 µm. The percentage of cleaved caspase‐3‐positive apoptotic cells among all tumor cells is presented H). Error bars represent mean ± SD. **p < 0.01, ***p < 0.001, ****p < 0.0001. I–L) Analysis of the therapeutic effects of CBP on tongue lesions induced by 4NQO in *Prmt1^Ctrl^* and *Prmt1^cKO^* mice. I) Experimental protocol for generation and drug treatment of *Prmt1^cKO^* mice. *Prmt1^cKO^* mice were generated by crossbreeding *Prmt1^fl/fl^* mice with *Krt14^Cre/ERT2^* mice. Following the induction of tongue injury with 4NQO over a 16‐week period, the mice were administered intraperitoneal injections of tamoxifen (120 mg kg^−1^, every other day) for 4 times at 24 weeks. This was followed by treatment with CBP (25 mg kg^−1^, i.p., twice/week) for four weeks prior to euthanasia. J) Representative images of visible tongue lesions across treatment groups are shown, with lesion areas outlined by black dashed lines. Quantification of the lesion areas in treated mice is also provided. Scale bar: 5 mm. K) Representative Hematoxylin and eosin (H&E) staining of lesions from treated mice is shown. Scale bar: 100 µm L) Images display cleaved caspase‐3 (red) and PRMT1 (green) in lesions, with nuclei counterstained with DAPI (blue). The percentage of cleaved caspase‐3‐positive apoptotic cells among all lesions is calculated. Scale bar: 20 µm. Error bars represent the mean ± SD, with significance levels of **p < 0.01, ***p < 0.001, and ****p < 0.0001.

Given that PDX models better mimic human tumors than CDX models, we further evaluated PRMT1 inhibition combined with CBP in PDX models. Fresh HNSCC tumors with high PRMT1 expression were selected for model establishment (**Figure** **2**A–C). Mice received intratumoral PRMT1 siRNA or control siRNA (siNC), along with intraperitoneal CBP or saline for three weeks. Consistent with CDX findings, PRMT1 knockdown significantly inhibited tumor growth, and tumors in the PRMT1 siRNA group showed heightened CBP sensitivity (Figure 2D–F; Figure S4G, Supporting Information). The combination therapy was well tolerated in vivo (Figure S4H, Supporting Information). Successful PRMT1 knockdown was confirmed via IHC (Figure 2G), and Ki67 and cleaved caspase‐3 staining patterns mirrored those in the CDX models (Figure 2G,H).

To investigate the role of PRMT1 in spontaneous HNSCC, we generated *Prmt1^cKO^* mice. In this model, PRMT1 was specifically deleted in the oral epithelium by crossing *Prmt1^fl/fl^* mice with *Krt14^Cre/ERT2^* mice, followed by tamoxifen‐induced gene deletion (Figure 2I; Figure S4I, Supporting Information). Mice were treated with 4NQO for 16 weeks to induce HNSCC, then administered tamoxifen (120 mg kg^−1^, every other day, four doses) starting at week 24, followed by CBP (25 mg kg^−1^, twice weekly for four weeks). *Prmt1^cKO^* mice exhibited fewer and smaller lesions than *Prmt1^Ctrl^* mice, and lesions were undetectable after CBP treatment by visual examination (Figure 2J). Histopathological grading showed reduced severity in *Prmt1^cKO^* mice, further diminished by CBP (Figure 2K). Additionally, cleaved caspase‐3 was elevated, indicating enhanced apoptosis, especially with CBP treatment (Figure 2L). In summary, PRMT1 silencing increases CBP sensitivity in HNSCC, highlighting PRMT1 as a promising therapeutic target.

### 2.3 IGF2BP2 Serves as a Functional Downstream Target of PRMT1

**Figure 3:**
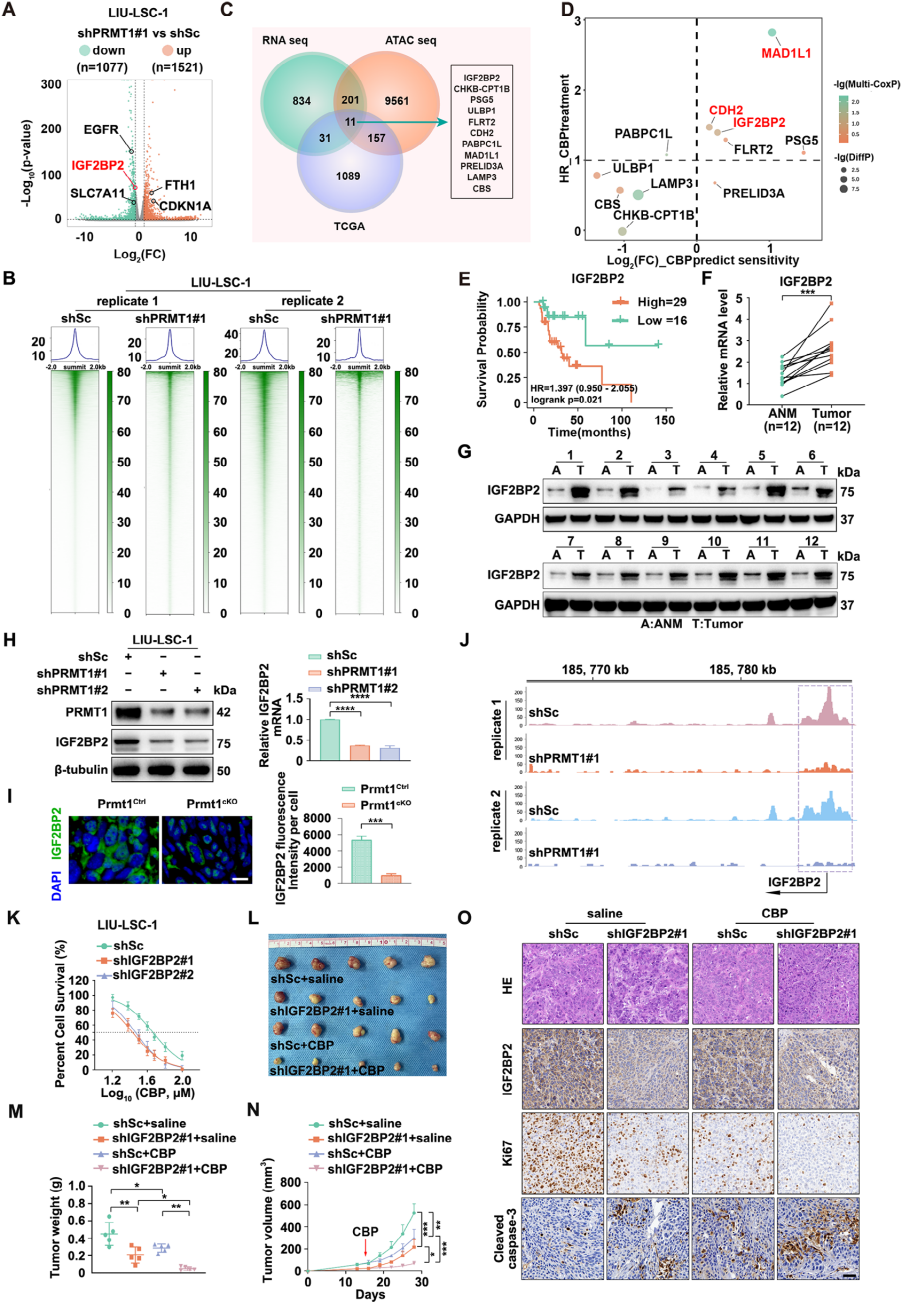
IGF2BP2 is a key functional downstream target of PRMT1. A,B) shPRMT1#1 LIU‐LSC‐1 cells and control cells were subjected to RNA‐seq and ATAC‐seq analysis. Panel A) shows a volcano plot of DEGs (|log_2_FC| > 1, *p* < 0.01). Panel B) shows heatmaps and enrichment plots displaying normalized read densities of ATAC‐seq signals following PRMT1 knockdown. Tracks are centered at the transcription start site (TSS) and extend ± 2 kb. C) Flowchart for screening the downstream targets of PRMT1. A Venn diagram illustrates 11 overlapping genes identified across three datasets: Dataset 1 consists of downregulated genes from RNA‐seq (shown panel A; log_2_FC < −1, *p* < 0.01), Dataset 2 includes genes annotated from differential peaks in ATAC‐seq (shown in panel B; log_2_FC < −1, *p* < 0.01), and Dataset 3 contains upregulated genes from the TCGA‐HNSCC dataset (log_2_FC > 2, *p* < 0.01). D) Relationship between the potential genes and CBP resistance in TCGA‐HNSCC patients. The scatter plot illustrates the hazard ratio (HR) from multivariate Cox regression for CBP treatment patients from TCGA‐HNSCC (*y*‐axis) and log_2_FC of CBP‐resistant (top 25%) versus CBP‐sensitive (bottom 25%) groups (*x*‐axis) based on OncoPredict R package. Genes with significant HR (HR > 1, *p* < 0.1) and altered expression are highlighted. The point color indicates the ‐log10 of multivariate Cox regression *p*‐values, and the point size reflects the −log10 of differential expression *p*‐values. E) Kaplan‐Meier survival analysis of TCGA‐HNSCC patients who received carboplatin treatment (*n* = 45), categorized into high (*n* = 29) and low (*n* = 16) IGF2BP2 expression groups based on optimal cutoff values. F,G) Paired ANM and HNSCC tissues (*n* = 12) were analyzed by qRT‐PCR F) and western blotting G), with error bars representing the mean ± SD. ***p < 0.001. H) PRMT1 knockdown LIU‐LSC‐1 cells and control cells were analyzed for IGF2BP2 expression using western blotting and qRT‐PCR. Error bars represent the mean ± SD. ****p < 0.0001. I) The expression of IGF2BP2 (green) and DAPI (blue) in tongue lesions from *Prmt1^Ctrl^* and *Prmt1^cKO^* mice was analyzed by IF. A representative image is shown in the left panel, and the quantitative analysis is presented in the right panel. Error bars represent mean ± SD. ****p < 0.0001. Scale bar: 20 µm. J) Visualization of the IGF2BP2 locus for ATAC‐seq signals using the Integrative Genomics Viewer (IGV). K) LIU‐LSC‐1 cells were transduced with lentivirus expressing shRNAs targeting IGF2BP2 (shIGF2BP2#1 and shIGF2BP2#2) or shSc. The IC50 values of CBP were determined using a CCK‐8 assay. L–O) Xenograft tumors derived from shIGF2BP2#1 LIU‐LSC‐1 cells and control cells were treated with CBP (25 mg kg^−1^, i.p., twice/week) or saline. Each group comprised 5 mice (*n* = 5). Tumor images L), tumor weights M), tumor volumes N), and representative IHC staining for IGF2BP2, Ki67, and cleaved caspase‐3 in xenograft tissues O) are presented. Scale bar: 50 µm. Error bars represent mean ± SD. *p < 0.05, **p < 0.01, ***p < 0.001.

To identify downstream targets of PRMT1, we performed RNA‐seq and assay for transposase‐accessible chromatin with high‐throughput sequencing (ATAC‐seq) on PRMT1 knockdown and control LIU‐LSC‐1 cells. RNA‐seq identified 2598 DEGs, including 1077 downregulated and 1521 upregulated genes (**Figure** **3**A; Table S2, Supporting Information). ATAC‐seq revealed significant changes in chromatin accessibility, with 9930 differential peaks distributed across promoters (28.58%), introns (25.34%), exons (23.35%), untranslated regions (14.59%), and intergenic regions (8.14%) (Figure 3B; Table S3, Supporting Information). Integrating downregulated genes and differential peak‐associated genes in PRMT1‐knockdown cells with upregulated genes from the TCGA‐HNSCC dataset (Table S4, Supporting Information) identified 11 overlapping candidates (Figure 3C). Kaplan‐Meier survival analysis and multivariate Cox regression in CBP‐treated TCGA‐HNSCC patients (*n* = 45) showed that high expression of MAD1L1, CDH2, PABPC1L, and IGF2BP2 correlated with poor prognosis (HR >1, *p* < 0.1) (Figure 3E; Figure S5A–J, Supporting Information). Given the absence of public HNSCC CBP resistance datasets, we used the OncoPredict R package to stratify TCGA‐HNSCC patients, revealing that IGF2BP2, CDH2, and MAD1L1 were significantly upregulated (p < 0.01) in the CBP‐resistant group (top 25%) compared to the CBP‐sensitive group (bottom 25%) (Figure 3D). Notably, only IGF2BP2 knockdown significantly enhanced CBP sensitivity in LIU‐LSC‐1 cells (Figure S5K–P, Supporting Information). Further analysis of 12 paired HNSCC and ANM tissues confirmed elevated IGF2BP2 mRNA and protein levels in tumors (Figure 3F,G), identifying IGF2BP2 as a key PRMT1 effector.

To explore the PRMT1‐IGF2BP2 relationship, we assessed IGF2BP2 expression in PRMT1 knockdown or overexpressing HNSCC cells. PRMT1 knockdown reduced IGF2BP2 levels in LIU‐LSC‐1 cells, while its overexpression increased IGF2BP2 in TU177 cells (Figure 3H; Figure S6A, Supporting Information). Prmt1‐deficient mouse lesions also showed significantly lower IGF2BP2 expression (Figure 3I). ATAC‐seq revealed that PRMT1 knockdown decreased chromatin accessibility at the *IGF2BP2* promoter (Figure 3J), indicating that IGF2BP2 is a direct downstream target of PRMT1 in HNSCC. Functional assays using IGF2BP2 knockdown or overexpression cell lines (Figure S6B,E, Supporting Information) demonstrated that IGF2BP2 silencing potentiated CBP‐induced suppression of cell survival and colony formation while enhancing apoptosis in LIU‐LSC‐1 cells (Figure 3K; Figure S6C,D, Supporting Information). In contrast, IGF2BP2 overexpression attenuated these CBP‐mediated effects in TU177 cells (Figure S6E–H, Supporting Information).

In vivo, xenograft assays confirmed that IGF2BP2 knockdown significantly inhibited tumor growth, with CBP further enhancing this effect (Figure 3L–N; Figure S5I, Supporting Information). IHC analysis showed that IGF2BP2 silencing reduced Ki67 expression and increased cleaved caspase‐3, indicating decreased proliferation and enhanced apoptosis, both of which were amplified by CBP treatment (Figure 3O). Importantly, no significant weight loss was observed in treated mice (Figure S6J, Supporting Information). In conclusion, like PRMT1, IGF2BP2 acts as a key driver of CBP resistance in HNSCC.

### 2.4 PRMT1 Enhances the Resistance of HNSCC Cells to CBP by Upregulating IGF2BP2

**Figure 4:**
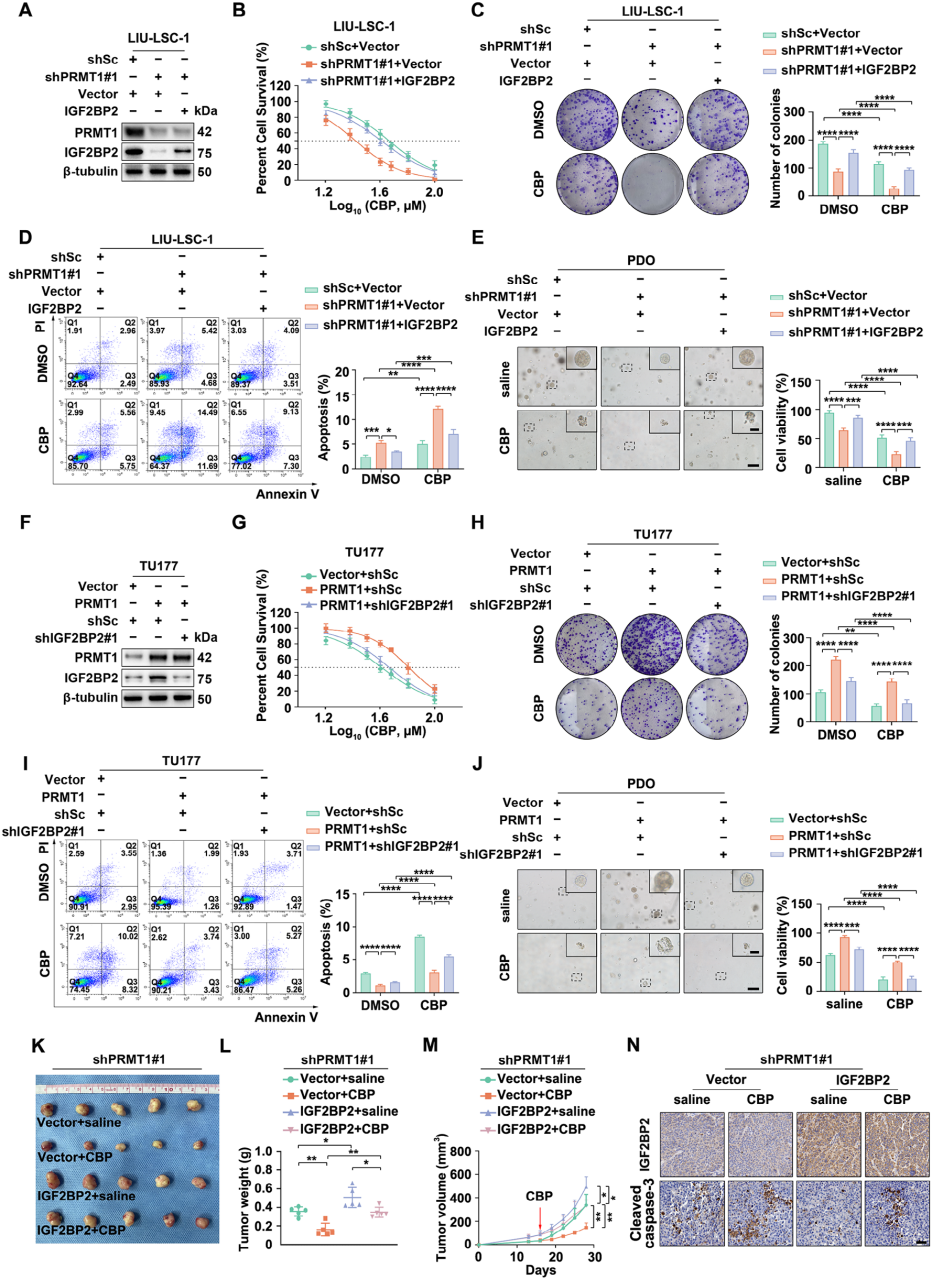
PRMT1 enhances the resistance of HNSCC cells to CBP by upregulating IGF2BP2. A–D) shPRMT1#1 LIU‐LSC‐1 cells and control cells were transduced with lentiviruses containing either a vector encoding human IGF2BP2 cDNA or an empty control vector. F–I) Lentiviruses expressing shIGF2BP2#1 or shSc were introduced into TU177 cells overexpressing PRMT1, as well as into control cells. A,F) Western blotting analysis was performed to evaluate the protein expression levels of PRMT1 and IGF2BP2. B,G) The CCK‐8 assay was conducted to determine the IC50 values of CBP. C,H) Cells were treated with CBP (45 µM, 48 h) or DMSO, and cell growth ability was assessed using colony formation assays. D,I) Cells were treated with CBP (45 µM, 24 h) or DMSO, and apoptosis was measured by flow cytometry. Error bars represent the mean ± SD. *p < 0.05, **p < 0.01, ***p < 0.001, ****p < 0.0001. E) Organoids with PRMT1 knockdown were transduced with lentiviruses carrying either a vector encoding human IGF2BP2 cDNA or an empty control vector. J) Organoids overexpressing PRMT1 and control organoids were transduced with lentiviruses expressing shIGF2BP2#1 or shSc. E,J) The indicated organoids were treated with CBP (45 µM) or saline for 48 h, and cell viability was assessed using the Cell‐Titer Glo‐3D assay. Representative phase contrast images are presented alongside data quantification. Scale bars: 200 µm (low magnification), 100 µm (high magnification). Error bars represent the mean ± SD. ***p < 0.001, ****p < 0.0001. K–N) The indicated shPRMT1#1 LIU‐LSC‐1 cells were subcutaneously injected into nude mice and subsequently treated with CBP (25 mg kg^−1^, i.p., twice/week) or saline on day 15. On day 28, the mice were euthanized, and the xenograft tissues were collected for further analysis. Tumor images K), tumor weights L), tumor volumes M), and representative IHC images demonstrating IGF2BP2 and cleaved caspase‐3 expression in xenograft tissues are presented N). Scale bar: 50 µm. Error bars represent the mean ± SD. *p < 0.05, **p < 0.01.

To investigate IGF2BP2's role in PRMT1 function, we overexpressed IGF2BP2 in PRMT1‐knockdown LIU‐LSC‐1 cells (**Figure** **4**A; Figure S7A, Supporting Information). Cytotoxicity assays, including IC50 measurements and clonogenic assays, showed that IGF2BP2 rescued the impaired proliferation and counteracted the increased CBP sensitivity caused by PRMT1 depletion (Figure 4B,C). Apoptosis assays further revealed that PRMT1 knockdown, especially with CBP treatment, significantly increased apoptosis in HNSCC cells, which was partially reversed by IGF2BP2 overexpression (Figure 4D).

We extended these findings to HNSCC PDOs, where PRMT1 knockdown inhibited growth and enhanced CBP sensitivity—effects counteracted by IGF2BP2 overexpression (Figure 4E; Figure S7B, Supporting Information). Conversely, IGF2BP2 knockdown in PRMT1‐overexpressing TU177 cells (Figure 4F; Figure S7C, Supporting Information) diminished the protective effects of PRMT1, reducing survival and colony formation under CBP treatment (Figure 4G,H). PRMT1 overexpression also suppressed CBP‐induced apoptosis, while IGF2BP2 knockdown restored CBP sensitivity (Figure 4I). Similarly, in HNSCC PDOs, IGF2BP2 knockdown reversed PRMT1‐induced CBP resistance (Figure 4J; Figure S7D, Supporting Information).

In vivo, a CDX tumor model was established by injecting IGF2BP2‐overexpressing shPRMT1#1 LIU‐LSC‐1 cells or control cells into nude mice. Mice received intraperitoneal CBP or saline for two weeks. IGF2BP2 overexpression promoted tumor growth and reduced CBP sensitivity without affecting body weight (Figure 4K–M; Figure S7E,F, Supporting Information). IHC confirmed that IGF2BP2 overexpression reversed CBP‐induced Ki67 reduction and cleaved caspase‐3 elevation in PRMT1‐knockdown tumors (Figure 4N; Figure S7G, Supporting Information). In conclusion, PRMT1 regulates IGF2BP2 expression, driving CBP resistance in HNSCC.

### 2.5 PRMT1 Recruits the SWI/SNF Chromatin Remodeling Complex by Binding to SMARCC1 and Subsequently Activating *IGF2BP2* Transcription

To assess whether PRMT1 upregulates IGF2BP2 independently of its enzymatic activity, we overexpressed three catalytically inactive PRMT1 mutants (G80R, M48A, M155A) in TU177 cells. Like wild‐type (WT) PRMT1, these mutants increased IGF2BP2 expression (Figure S8A, Supporting Information). Furthermore, treatment with the PRMT1‐specific inhibitor iPRMT1 effectively reduced H4R3me2a levels, a PRMT1‐catalyzed histone mark, but had minimal impact on IGF2BP2 expression in LIU‐LSC‐1 cells (Figure S8B, Supporting Information). These findings suggest PRMT1 regulates IGF2BP2 independently of its catalytic function.

**Figure 5:**
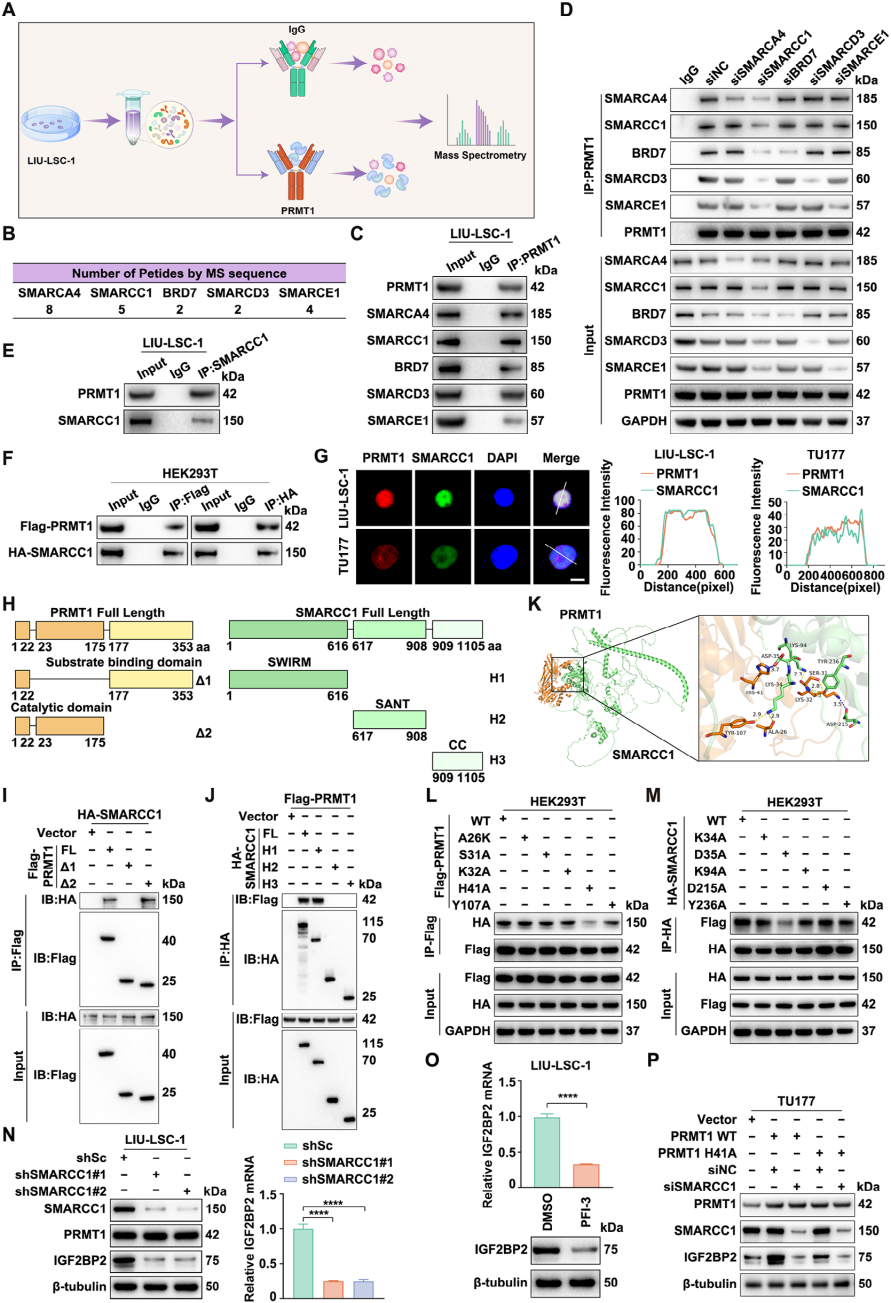
PRMT1 recruits the SWI/SNF chromatin remodeling complex by interacting with SMARCC1 and then activates the transcription of IGF2BP2. A) Workflow diagram of IP‐MS experiments utilizing PRMT1 antibodies. B) Number of peptides from the subunits of the SWI/SNF complex identified by MS analysis. C) PRMT1 was immunoprecipitated in LIU‐LSC‐1 cells, followed by immunoblotting with indicated antibodies. D) LIU‐LSC‐1 cells were transfected with siRNA targeting SMARCA4, SMARCC1, BRD7, SMARCD3, SMARCE1, or negative control (siNC) for 48 h. The cells were then immunoprecipitated using PRMT1 antibodies, followed by immunoblotting with the indicated antibodies. E) Immunoprecipitation (IP) of SMARCC1 was performed in LIU‐LSC‐1 cells, followed by immunoblotting with PRMT1 antibodies. F) HEK293T cells were co‐transfected with Flag‐PRMT1 and HA‐SMARCC1 plasmids, followed by IP for Flag and HA, respectively. G) Representative IF images showing the co‐localization of PRMT1 and SMARCC1 in HNSCC cells. The intensity profiles of PRMT1 and SMARCC1 along the white line were plotted. Scale bars, 8 µm. H) Schematic diagram illustrating the construction of full‐length (FL) and truncated PRMT1 and SMARCC1 proteins. I) Flag‐tagged FL PRMT1 or the indicated truncation mutants were co‐expressed with HA‐tagged SMARCC1 in HEK293T cells. After IP using Flag antibodies, the samples were subjected to immunoblot analysis to detect HA‐SMARCC1 with an anti‐HA antibody. J) HEK293T cells were co‐expressed with HA‐tagged SMARCC1 FL or the indicated truncation mutants and Flag‐tagged PRMT1. SMARCC1 FL and truncated proteins were then immunoprecipitated using anti‐HA antibodies, followed by immunoblot analysis to detect Flag‐PRMT1 with an anti‐Flag antibody. K) Molecular docking model of SMARCC1 interacting with PRMT1. L) Flag‐PRMT1 WT or various point mutants were co‐expressed with HA‐SMARCC1 in HEK293T cells. Flag‐PRMT1 was then immunopurified using Flag beads, followed by immunoblotting with an HA antibody. M) Co‐expression of HA‐SMARCC1 WT or various point mutants with Flag‐PRMT1 was performed in HEK293T cells. Following this, HA‐SMARCC1 was immunopurified using HA beads, and immunoblotting was conducted with a Flag antibody. N) LIU‐LSC‐1 cells were transduced with lentivirus expressing shRNAs targeting SMARCC1 (shSMARCC1#1, shSMARCC1#2) or shSc. O) LIU‐LSC‐1 cells were treated with PFI‐3 (10 µM) or DMSO for 48 h. N,O) The expression of IGF2BP2 was assessed by western blotting and qRT‐PCR. Error bars represent mean ± SD. ****p <0.0001. P) TU177 cells overexpressing either PRMT1 WT or PRMT1 H41A were transfected with siRNA targeting SMARCC1 or siNC for 48 h. Cell lysates were then subjected to immunoblotting with the indicated antibodies.

To explore the underlying mechanism, we performed immunoprecipitation‐based mass spectrometry (IP‐MS) to identify PRMT1‐interacting proteins (**Figure** **5**A). Among them, SMARCA4 and other SWI/SNF complex subunits (SMARCC1, BRD7, SMARCD3, SMARCE1) stood out (Figure 5B; Table S5, Supporting Information). Co‐immunoprecipitation (Co‐IP) assays confirmed that PRMT1 interacts with these subunits in LIU‐LSC‐1 cells (Figure 5C). Further, siRNA knockdown experiments revealed that only SMARCC1 depletion disrupted PRMT1's association with the SWI/SNF complex, identifying SMARCC1 as its key binding partner (Figure 5D). Co‐IP in LIU‐LSC‐1 and HEK293T cells further validated the PRMT1‐SMARCC1 interaction (Figure 5E,F), and a GST pull‐down assay confirmed their direct binding in vitro (Figure S9A, Supporting Information). IF assays showed their nuclear co‐localization in LIU‐LSC‐1 and TU177 cells (Figure 5G), suggesting PRMT1 recruits the SWI/SNF complex via SMARCC1.

To map their interaction domains, we generated truncated mutants (Figure 5H). Co‐IP revealed that PRMT1 interacts with SMARCC1 through its catalytic domain (Figure 5I), while SMARCC1 binds PRMT1 via its SWIRM domain (Figure 5J). Molecular docking predicted key interaction residues: A26, S31, K32, H41, Y107 in PRMT1 and K34, D35, K94, D215, Y236 in SMARCC1 (Figure 5K). Mutation of H41 in PRMT1 and D35 in SMARCC1 disrupted their interaction, as confirmed by Co‐IP (Figure 5L,M).

Despite this interaction, PRMT1 had no direct effect on SMARCC1 expression at either the mRNA or protein level (Figure S9B,C, Supporting Information). However, SMARCC1 knockdown significantly reduced IGF2BP2 expression in LIU‐LSC‐1 cells (Figure 5N). Inhibiting SWI/SNF complex activity with PFI‐3 similarly decreased IGF2BP2 levels (Figure 5O). Importantly, WT PRMT1, but not the H41A mutant lacking SMARCC1 binding, upregulated IGF2BP2 expression. Additionally, SMARCC1 knockdown reversed PRMT1‐induced IGF2BP2 upregulation (Figure 5P). Chromatin immunoprecipitation (ChIP)‐qPCR and sequential chromatin immunoprecipitation (ChIP‐reChIP) confirmed the co‐occupancy of PRMT1 and SMARCC1 at the *IGF2BP2* promoter (Figure S9D,E, Supporting Information), indicating PRMT1 recruits the SWI/SNF complex via SMARCC1 to activate *IGF2BP2* transcription.

To identify transcription factors (TFs) involved in this regulation, we performed JASPAR TF binding site enrichment analysis, revealing 85 potential TFs binding at the *IGF2BP2* promoter (Table S6, Supporting Information). Integrating hTFtarget, GTRD, and ChIPBase datasets narrowed this to 17 candidates, of which four (E2F4, ETV4, ZNF263, E2F6) correlated with IGF2BP2 expression in TCGA‐HNSCC (*r* > 0.25, *p* < 0.05) (Figure S10A,B, Supporting Information). Among them, only ETV4 knockdown significantly reduced IGF2BP2 levels in LIU‐LSC‐1 cells (Figure S10C–F, Supporting Information). ChIP‐qPCR showed PRMT1 or SMARCC1 knockdown reduced ETV4 occupancy at the *IGF2BP2* promoter (Figure S10G, Supporting Information), and ETV4 depletion reversed PRMT1‐induced IGF2BP2 upregulation (Figure S10H, Supporting Information). These results suggest that ETV4 is essential for PRMT1‐SWI/SNF complex‐mediated IGF2BP2 activation.

### 2.6 PBX2 Transcriptionally Activates PRMT1 and Subsequently Upregulates the Expression of IGF2BP2

**Figure 6:**
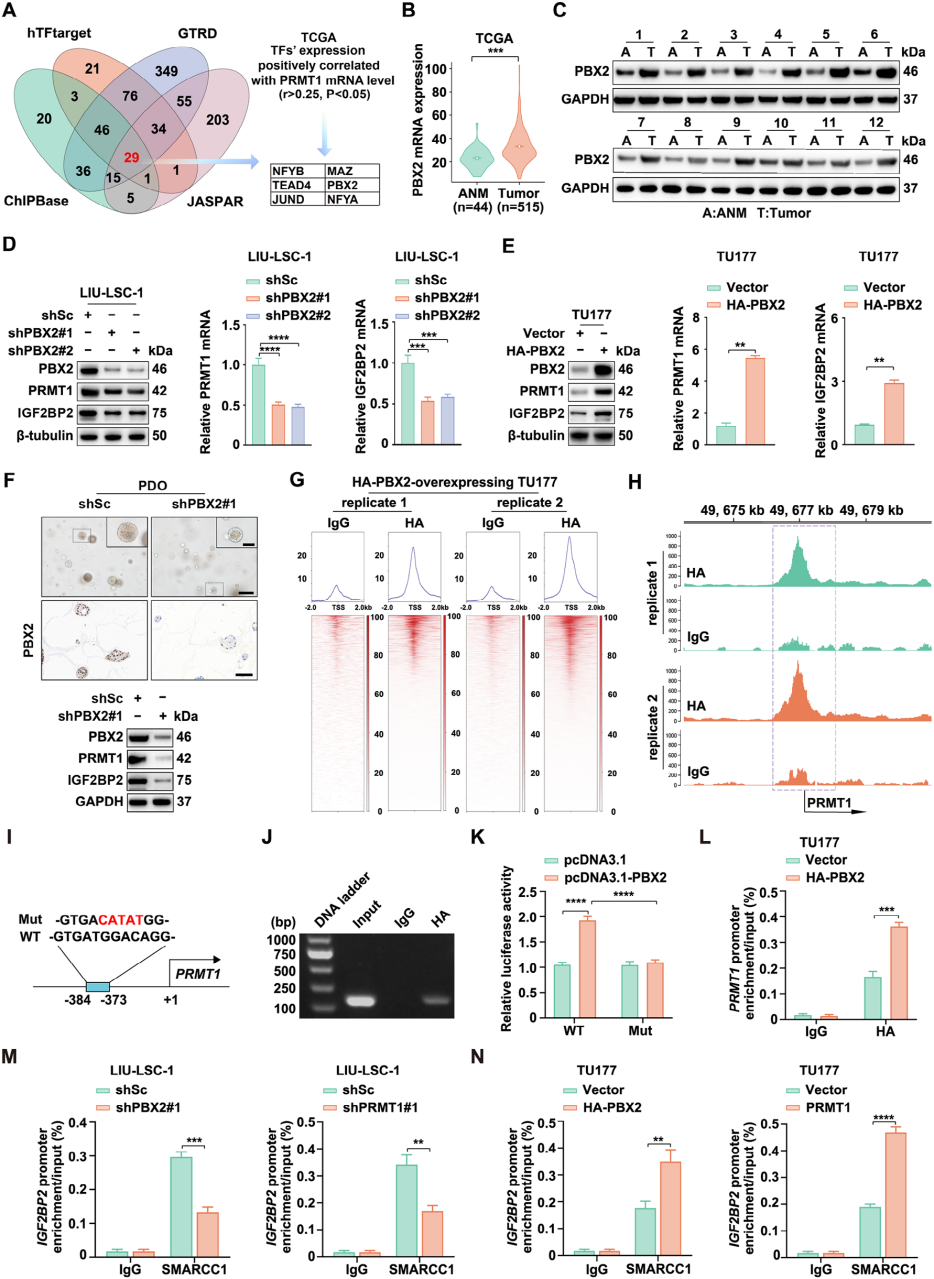
PBX2 transcriptionally activates PRMT1 gene expression in HNSCC cells. A) Flowchart for screening six potential TFs of PRMT1. The Venn diagram displays 29 TFs identified from four databases. A correlation analysis of these 29 TFs with the *PRMT1* mRNA levels was performed using the TCGA‐HNSCC dataset (R > 0.25, *p* < 0.05). B) Analysis of PBX2 expression in ANM (*n* = 44) compared to tumor tissues (*n* = 515) derived from TCGA‐HNSCC dataset. C) Paired ANM and HNSCC tissues (*n* = 12) were analyzed using western blotting with PBX2 antibodies. D) LIU‐LSC‐1 cells were transfected with lentiviral shRNAs targeting PBX2 (shPBX2#1, shPBX2#2) or a scrambled sequence (shSc). E) TU177 cells were transduced with either a control vector or lentiviruses expressing HA‐PBX2. D,E) The expression levels of PRMT1 and IGF2BP2 were assessed using western blotting (left panels) and qRT‐PCR (right panels). Error bars represent the mean ± SD. **p < 0.01, ***p < 0.001, ****p < 0.0001. F) HNSCC organoids were infected with lentiviral shRNAs targeting PBX2 (shPBX2#1) or shSc. Representative phase‐contrast images of the organoids are shown in the upper panels. Scale bars: 200 µm (low magnification) and 100 µm (high magnification). Additionally, representative IHC images depict PBX2 expression in the middle panels, with a scale bar of 50 µm. PBX2 protein levels were assessed using western blotting, shown in the lower panels. G) Heatmap showing CUT&Tag counts across distinct HA‐PBX2 binding peaks in HA‐PBX2 overexpressing TU177 cells. H) The HA‐PBX2 binding peak in the *PRMT1* gene was visualized using IGV. I) Schematic representation of the potential PBX2 binding site within the promoter region of the PRMT1 gene. J) ChIP‐PCR assay analyzing PBX2 enrichment within the promoter region of the *PRMT1* gene in HA‐PBX2‐overexpressing TU177 cells. K) HEK293T cells were co‐transfected with PRMT1wt‐Luc or pPRMT1mut‐Luc, along with pcDNA3.1‐PBX2 or the empty vector pcDNA3.1, and the internal control plasmid pRL‐TK. Relative luciferase activity was measured 24 h after transfection. Error bars represent the mean ± SD. ****p < 0.0001. L) ChIP analysis was performed on HA‐PBX2‐overexpressing TU177 cells and control cells using HA antibodies or control rabbit IgG. qPCR was used to amplify regions surrounding the putative PBX2 binding site in the promoter region of the *PRMT1* gene. Error bars represent the mean ± SD. ***p < 0.001. M,N) ChIP analysis was conducted on shPBX2#1 or shPRMT1#1 LIU‐LSC‐1 cells M), HA‐PBX2‐ or PRMT1‐overexpressing TU177 cells N), and their corresponding control cells, using antibodies against SMARCC1, or control rabbit IgG. qPCR was performed to amplify the promoter region (−800 bp to −665 bp) of the *IGF2BP2* gene. Error bars represent the mean ± SD. **p < 0.01, ***p < 0.001, ****p < 0.0001.

To investigate the mechanisms underlying the elevated expression of PRMT1 during HNSCC progression, bioinformatics analyses were performed using four publicly available databases. This analysis identified 29 potential TFs (**Figure** **6**A). To narrow down the candidate TFs, we examined the correlation between their mRNA levels and PRMT1 expression in the TCGA‐HNSCC dataset. PRMT1 expression was positively correlated with six TFs: NFYB, MAZ, TEAD4, PBX2, JUND, and NFYA (*r* > 0.25, *p* < 0.05; Figure 6A; Table S6, Supporting Information). Subsequent siRNA‐mediated knockdown of these six TFs revealed that only PBX2 depletion significantly reduced both PRMT1 mRNA and protein levels (Figure S11A–F, Supporting Information). These findings suggest that PBX2 may regulate PRMT1 expression.

Analysis of the TCGA‐HNSCC dataset showed significant upregulation of PBX2 in HNSCC tissues compared to ANM tissues (Figure 6B). Additionally, analysis of 12 paired HNSCC and ANM samples confirmed substantial elevation of PRMT1 expression in HNSCC tissues (Figure 6C). Lentivirus‐mediated shRNA knockdown of PBX2 led to a marked reduction in both PRMT1 and IGF2BP2 expression (Figure 6D). Conversely, PBX2 overexpression increased PRMT1 and IGF2BP2 levels at both the mRNA and protein levels (Figure 6E). Consistent with these findings, PDO experiments demonstrated that PBX2 depletion significantly reduced PRMT1 and IGF2BP2 expression in PDOs (Figure 6F). Collectively, these results support the role of PBX2 in regulating PRMT1 expression in HNSCC cells.

To further investigate the mechanism by which PBX2 regulates PRMT1, genome‐wide cleavage under targets and tagmentation (CUT&Tag) analysis was performed in HA‐PBX2‐overexpressing TU177 cells using HA‐specific antibodies (Figure 6G; Table S7, Supporting Information). CUT&Tag sequencing revealed a pronounced peak increase within the *PRMT1* gene in the HA‐PBX2 group relative to the IgG group (Figure 6H). Analysis of the increased peak region (19q13.2: 49 676 396–49 677 556) in the CUT&Tag sequencing results of the human *PRMT1* gene predicted a PBX2 binding site (‐384/‐373; GTGATGGACAGG) using JASPAR (Figure 6I). ChIP‐PCR confirmed PBX2 enrichment at this site, highlighting its role in regulating *PRMT1* transcription (Figure 6J).

Next, the promoter region (−426/+153) of the *PRMT1* gene was cloned into the pGL3‐basic luciferase reporter vector to assess PBX2's effect on its activity. PBX2 overexpression significantly increased the luciferase activity of the WT construct, while mutation of the binding site abolished this effect (Figure 6K). ChIP‐qPCR analysis further validated increased PBX2 occupancy at the *PRMT1* promoter region in HA‐PBX2‐overexpressing cells (Figure 6L). Additionally, ChIP‐qPCR revealed that knockdown of PBX2 or PRMT1 reduced SMARCC1 occupancy on the *IGF2BP2* promoter (Figure 6M), while PBX2 or PRMT1 overexpression enhanced SMARCC1 occupancy (Figure 6N). These findings suggest that PBX2 directly binds to the *PRMT1* gene, promoting its transcription, and upregulates IGF2BP2 expression by recruiting the SWI/SNF complex.

### 2.7 Activation of the PBX2‐PRMT1‐SWI/SNF‐IGF2BP2 Signaling Pathway is Associated with Malignant Progression and Poor Prognosis of HNSCC

**Figure 7:**
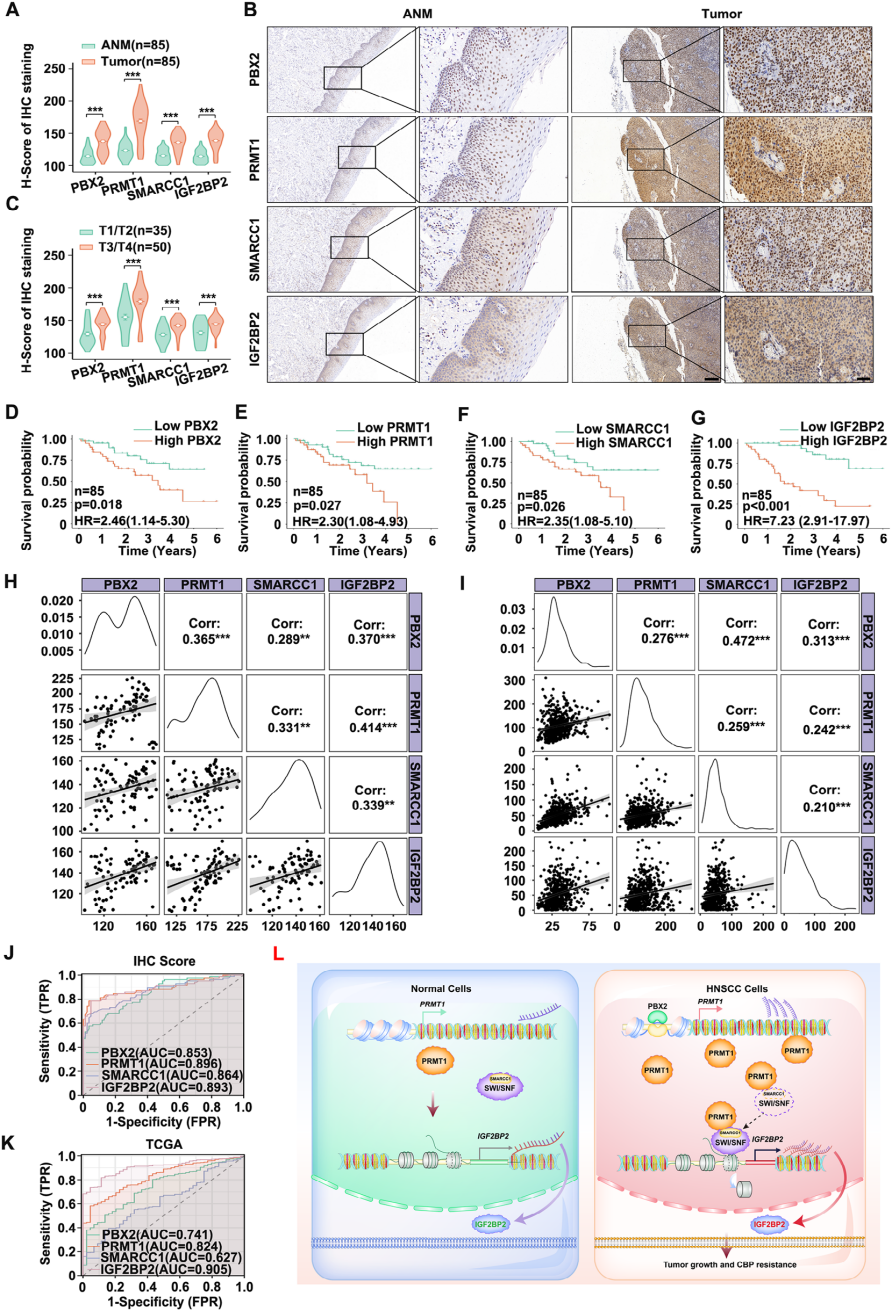
Activation of the PBX2‐PRMT1‐SWI/SNF‐IGF2BP2 signaling cascade is associated with malignant progression and poor prognosis in HNSCC. A, B) IHC staining was performed to evaluate the expression levels of PBX2, PRMT1, SMARCC1, and IGF2BP2 in paired ANM and HNSCC tissues (*n* = 85). H‐score analysis was conducted to quantify the staining A), and representative IHC images are presented B). Scale bars: 200 µm (low magnification) and 50 µm (high magnification). Error bars represent the mean ± SD. ***p < 0.001. C) H‐score analysis comparing IHC staining between T1/T2 and T3/T4 HNSCC tissues. Error bars represent the mean ± SD. ***p < 0.001. D–G), K–M) analysis of the association between overall survival and PBX2 D), PRMT1 E), SMARCC1 F), and IGF2BP2 G) protein levels in 85 HNSCC patients. Based on the median H‐score of IHC staining, the expression levels of PBX2, PRMT1, SMARCC1, and IGF2BP2 were divided into high (*n* = 42) and low (*n* = 43) subgroups. H,I) The correlation of expression levels among PBX2, PRMT1, SMARCC1, and IGF2BP2 in HNSCC tissues from the 85 cases H) and the TCGA‐HNSCC dataset I) was analyzed. Spearman's correlation coefficient values are indicated in the upper right corner. Scatterplot matrices with fitted trend lines for the respective genes are presented in the lower left corner. Error bars represent the mean ± SD. **p < 0.01, ***p < 0.001. J,K) ROC curves were generated to evaluate the diagnostic utility of PBX2, PRMT1, SMARCC1, and IGF2BP2 expression levels based on H‐scores from 85 HNSCC tissues J) and the TCGA‐HNSCC dataset K). L) A schematic diagram illustrating the critical role of the activated PBX2‐PRMT1‐SWI/SNF‐IGF2BP2 signaling pathway in promoting tumor growth and conferring resistance to CBP in HNSCC.

IHC staining was employed to analyze the expression levels of PBX2, PRMT1, SMARCC1, and IGF2BP2 in a retrospective cohort of 85 clinicopathologically characterized HNSCC cases. The cohort included 14 cases of tumor stage T1, 21 cases of T2, 23 cases of T3, and 27 cases of T4 (Table S8, Supporting Information). Protein expression levels were quantified using H‐scores, revealing significantly higher levels of PBX2, PRMT1, SMARCC1, and IGF2BP2 in HNSCC tissues compared to ANM tissues (**Figure** **7**A,B). IHC analysis of these proteins showed significantly higher H‐scores in advanced T stages (T3/T4) compared to early stages (T1/T2), suggesting that the elevated expression is associated with tumor progression (Figure 7C).

Furthermore, based on the median expression levels of PBX2, PRMT1, SMARCC1, and IGF2BP2, the 85 HNSCC cases were categorized into high and low‐expression groups. As illustrated in Figure 7D–G, elevated levels of these proteins were associated with poorer prognosis in HNSCC patients. Spearman's correlation analysis further revealed a positive correlation among their expression levels in the 85 cases (Figure 7H). This observation was consistent with findings from the TCGA‐HNSCC dataset and GSE130605, where similar positive correlations were also noted (Figure 7I; Figure S12, Supporting Information).

Next, receiver operating characteristic (ROC) curves were generated based on the expression levels of PBX2, PRMT1, SMARCC1, and IGF2BP2 in the 85 HNSCC samples, yielding area under the curve (AUC) values of 0.853, 0.896, 0.864, and 0.893, respectively (Figure 7J). Similar ROC curve analysis using the TCGA‐HNSCC dataset produced AUC values of 0.741, 0.824, 0.627, and 0.905 for PBX2, PRMT1, SMARCC1, and IGF2BP2, respectively (Figure 7K). These consistent findings suggest that the expression of PBX2, PRMT1, SMARCC1, and IGF2BP2 in tumor tissues could serve as reliable diagnostic markers for HNSCC due to their high sensitivity and specificity. In conclusion, the activated PBX2‐PRMT1‐SWI/SNF‐IGF2BP2 signaling pathway likely plays a critical role in the progression and prognosis of HNSCC (Figure 7L).

## 3 Discussion

Accumulating evidence suggests that PRMT1 is aberrantly overexpressed in multiple cancers, driving proliferation, invasion, metastasis, and chemoresistance.^[^ ^12^, ^13^, ^15^ ^]^ For example, PRMT1 is elevated in breast cancer tissues, and its inhibition suppresses tumor growth.^[^ ^16^ ^]^ Similarly, in esophageal squamous cell carcinoma (ESCC), PRMT1 overexpression correlates with poor prognosis and enhances stem‐like properties, chemoresistance, and tumorigenicity.^[^ ^12b^ ^]^ However, its precise role in HNSCC remains unclear. Here, we demonstrate that PRMT1 is highly expressed in HNSCC and is associated with poor patient outcomes. Functionally, PRMT1 promotes tumor growth and confers CBP resistance across multiple preclinical models, including HNSCC cell lines, CDX, PDO, PDX, and *Prmt1^cKO^* mouse models, suggesting that targeting PRMT1 in combination with CBP could be a promising therapeutic strategy.

While our study highlights the therapeutic potential of PRMT1 inhibition combined with CBP in multiple preclinical models, including PDX, we acknowledge inherent limitations of PDX models.^[^ ^17^ ^]^ Despite their ability to better replicate human tumor heterogeneity compared to cell lines, PDX models exhibit inter‐sample variability due to factors such as the tumor microenvironment, patient‐specific characteristics, and engraftment in immunodeficient mice, which may affect treatment response consistency. Nevertheless, our PDX cohorts consistently showed tumor growth inhibition and increased sensitivity to combination therapy, supporting the clinical relevance of targeting PRMT1 in HNSCC. Future studies should expand PDX models with varying PRMT1 expression to validate these findings across diverse HNSCC subtypes.

PRMT1 is responsible for 85% of global arginine methyltransferase activity and catalyzes the formation of H4R3me2a, a histone modification linked to transcriptional activation.^[^ ^18^ ^]^ It has been implicated in tumor progression by regulating key oncogenic genes.^[^ ^19^ ^]^ For instance, PRMT1 enhances neuroblastoma cell survival by upregulating ATF5 and drives colorectal cancer progression by upregulating EGFR and TNS4 expression through H4R3me2a.^[^ ^12f^ ^]^ However, its downstream effectors in HNSCC remain unclear. In this study, we identify IGF2BP2 as a novel downstream target of PRMT1 in HNSCC through integrated analysis of RNA‐seq, ATAC‐seq, and TCGA‐HNSCC data. Functional studies demonstrate that PRMT1 regulates IGF2BP2 expression, which in turn mediates PRMT1‐driven oncogenic effects, including tumor proliferation, apoptosis resistance, and CBP sensitivity. Clinically, IGF2BP2 is significantly upregulated in HNSCC, correlates with poor prognosis, and strongly associates with PRMT1 expression, supporting its role as a key effector in PRMT1‐driven tumorigenesis.^[^ ^20^ ^]^ Beyond HNSCC, IGF2BP2, an RNA‐binding protein involved in mRNA localization, stability, and translational control, is also upregulated in hepatocellular carcinoma (HCC) and lung cancer, where it plays a pivotal role in tumorigenesis.^[^ ^21^ ^]^ Notably, PRMT1 is elevated in these cancers as well.^[^ ^22^ ^]^ Our findings further reveal that PRMT1 regulates IGF2BP2 expression in HCC and lung adenocarcinoma (LUAD) cell lines (Figure S13A–D, Supporting Information), suggesting that the PRMT1‐IGF2BP2 axis may represent a broader oncogenic mechanism across multiple malignancies.

PRMT1 plays a pivotal role in tumor progression, primarily by methylating target proteins to modulate their activity or stability.^[^ ^23^ ^]^ For example, PRMT1 promotes HCC growth by methylating PHGDH, enhancing its enzymatic activity, and driving serine synthesis and oxidative stress resistance.^[^ ^22^, ^24^ ^]^ Similarly, PRMT1 stabilizes DDX3 through arginine methylation, preserving mitochondrial homeostasis and facilitating breast cancer metastasis.^[^ ^23^ ^]^ However, its non‐enzymatic functions remain largely unexplored. In this study, we reveal a novel, methyltransferase‐independent role of PRMT1 in upregulating IGF2BP2 expression in HNSCC, as well as in HCC and lung cancer cells (Figure S13C,D, Supporting Information). This study expands the understanding of PRMT1 beyond its enzymatic activity. Notably, previous research has shown that PRMT1 interacts with the nuclear receptor Nur77, enhancing its protein levels and transcriptional activity independently of methylation.^[^ ^25^ ^]^ These findings collectively highlight PRMT1's non‐enzymatic role in gene regulation, providing new insights into its function in cancer biology. Our further investigation revealed that PRMT1 recruits the SWI/SNF chromatin remodeling complex via direct binding to SMARCC1, thereby promoting *IGF2BP2* transcription.^[^ ^26^ ^]^ SWI/SNF‐mediated chromatin remodeling is an emerging mechanism of gene regulation by nuclear proteins.^[^ ^27^ ^]^ For instance, KLF4 recruits the SWI/SNF complex in response to shear stress to enhance endothelial gene expression, while chromatin‐bound cGAS utilizes SWI/SNF to regulate glutaminolysis and DNA replication.^[^ ^28^ ^]^ Our study identifies PRMT1 as another key modulator leveraging SWI/SNF to facilitate oncogenic gene expression.

Given the central role of SWI/SNF in PRMT1‐driven transcription, targeting this complex presents a promising therapeutic strategy for PRMT1‐overexpressing tumors. Small‐molecule inhibitors of SMARCA2/4 and emerging PROTAC‐based degradation approaches have shown potential in disrupting SWI/SNF function.^[^ ^29^ ^]^ Furthermore, our findings offer an explanation for the limited efficacy of PRMT1 enzymatic inhibitors in clinical trials, suggesting that PRMT1's oncogenic effects extend beyond methylation. A combination strategy targeting both PRMT1 enzymatic activity and SWI/SNF recruitment may provide a more effective therapeutic approach for PRMT1‐driven cancers.

Considering PRMT1 overexpression in various human malignancies, understanding its regulatory mechanisms is crucial.^[^ ^30^ ^]^ PRMT1 undergoes proteasome‐mediated degradation; for instance, TRIM48 facilitates its ubiquitination, promoting oxidative stress‐induced cell death, while FBXO7‐mediated degradation suppresses serine synthesis and inhibits HCC growth.^[^ ^11^, ^22^, ^31^ ^]^ Additionally, non‐coding RNAs such as miR‐503 and LINC01431 modulate PRMT1 expression, yet its transcriptional regulation in tumors remains largely unexplored. Here, we identify PBX2 as a previously unrecognized transcriptional regulator of PRMT1. PBX2, known to drive tumor progression by modulating oncogenes and non‐coding RNAs,^[^ ^36^ ^]^ had unclear direct targets in HNSCC. Our findings establish PRMT1 as a novel PBX2 effector, providing key insights into its transcriptional control and suggesting that targeting the PBX2‐PRMT1 axis may offer a promising therapeutic strategy.

Since PRMT1 promotes tumor growth beyond its methyltransferase activity, conventional enzymatic inhibitors may have limited efficacy. Consequently, alternative approaches such as disrupting PRMT1's protein‐protein interactions or using targeted degradation strategies, like PROTAC, should be further explored. While TFs are traditionally difficult to target, developing small molecules or peptide inhibitors—potentially guided by computational modeling^[^ ^32^ ^]^—could offer a promising strategy to interfere with PBX2‐DNA binding or its interactions with co‐regulators. Supporting this, our CUT&Tag analysis identified a broader set of PBX2‐bound genes (Table S7, Supporting Information), suggesting its involvement in an extensive transcriptional network in HNSCC. Moreover, TCGA data analysis reveals significant upregulation of both PBX2 and PRMT1 in HCC and kidney renal papillary cell carcinoma, with a strong positive correlation (Figure S13E,F, Supporting Information). These findings indicate that the PBX2‐PRMT1 axis may play a crucial role in tumorigenesis across various malignancies, underscoring its potential as a therapeutic target.

In summary, we identify the PBX2‐PRMT1‐SWI/SNF‐IGF2BP2 axis as a key driver of tumor growth and CBP resistance in HNSCC, revealing non‐methyltransferase functions of PRMT1 and its potential as a therapeutic target to enhance CBP efficacy.

## 4 Experimental Section

### Sample Collection

HNSCC samples for western blotting, qRT‐PCR, and IHC analysis were collected from patients at the First Affiliated Hospital of Anhui Medical University from 2014 to 2020. Fresh tumor tissues were obtained for the establishment and characterization of PDO and PDX models. Pathological diagnoses were confirmed by at least two experienced pathologists, and written informed consent was obtained from each patient prior to tissue collection. All procedures were conducted in accordance with protocols approved by the hospital's Ethics Committee. Detailed clinical information‐including age, gender, pathological grade, TNM stage, lymph node involvement, and local invasion‐can is provided in Tables S8 and S9 (Supporting Information), while clinical data for patients involved in PDO and PDX model development are summarized in Table S10 (Supporting Information).

### Cell Line and Cell Culture

The FaDu cell line, originating from HNSCC, PLC/PRF/5 cell line from HCC, NCI‐H1975 cell line from LUAD, and HEK293T cells were acquired from the American Type Culture Collection (ATCC, VA, USA). The TU686 HNSCC cell line was obtained from the BeNa Culture Collection in Beijing, China, whereas Huh‐7 cell line, PC‐9 LUAD cell line, TU212, and human normal oral keratinocyte (NOK) cells were sourced from Otwo Biotech in Shenzhen, China. The human HNSCC cell lines TU177 and LIU‐LSC‐1 have been previously described and characterized.^[^ ^33^ ^]^ PC‐9 cells, NCI‐H1975 cells, and other HNSCC cell lines were cultured in 1640 medium (Gibco, NY, USA) supplemented with 10% fetal bovine serum (FBS, Biological Industries) and penicillin (100 U mL^−1^)/streptomycin (100 µg mL^−1^) (Beyotime, Shanghai, China). PLC/PRF/5 cells, Huh‐7 cells, and HEK293T cells were cultured in Dulbecco's Modified Eagle's Medium (DMEM) (Gibco) with the same composition at 37 °C in a humidified incubator containing 5% CO_2_. Additional details on the origins and culture conditions of the cell lines are provided in Table S11 (Supporting Information).

### Antibodies and Reagents

Antibodies used in this study are listed in Table S12 (Supporting Information). CBP, Z‐VAD‐FMK, Chloroquine, iPRMT1, and PFI‐3 were procured from MedChemExpress (NJ, USA), while puromycin, dimethyl sulfoxide (DMSO), and tamoxifen were obtained from Sigma‐Aldrich (MO, USA). Additionally, 4‐nitroquinoline N‐oxide (4NQO) was sourced from Santa Cruz Biotechnology (CA, USA).

### RNA Interference

RNA interference experiments were conducted in accordance with the manufacturer's protocol. siRNA oligonucleotides were synthesized by GenePharma (Shanghai, China). Cell transfections were performed using Lipofectamine RNAiMax (Thermo Fisher Scientific, MA, USA) when cell confluence reached 50–60%. The target sequences for siRNAs are detailed in Table S13 (Supporting Information).

### qRT‐PCR

Total RNA was extracted using TRIzol reagent (Invitrogen, CA, USA), and its quality and concentration were assessed using a NanoDrop 2000 spectrophotometer (Thermo Fisher Scientific). A total of 1 µg RNA was used to synthesize first‐strand cDNA with the RevertAid First Strand cDNA Synthesis Kit (Thermo Fisher Scientific). qRT‐PCR was performed on a LightCycler 96 system (Roche, Switzerland) using SYBR Premix Ex Taq II (TaKaRa, Kyoto, Japan), following the manufacturer's protocols. Gene expression levels were normalized to β‐actin using the 2^−ΔΔCt^ method. The primers used for qRT‐PCR were designed and synthesized by Sangon Biotech (Shanghai, China), and are listed in Table S14 (Supporting Information).

### Cell Viability

Cell viability and IC50 values were determined using the Cell Counting Kit‐8 (CCK‐8) assay (Beyotime), following the manufacturer's protocol. HNSCC cells were seeded at a density of 1 × 10^4^ cells per well in 96‐well plates. After incubating for 12 h, the cells were treated with CBP at the indicated concentrations for 72 h. 10% CCK‐8 solution was added to each well, and cells were incubated at 37 °C for 2 h. Absorbance at 450 nm was measured using a microplate reader, and cell viability was calculated as described in the manufacturer's instructions. IC50 values were derived based on the relative cell viability percentage compared to the control, using the formula: % cell viability = [(absorbance of experimental well − absorbance of blank) / (absorbance of untreated control well − absorbance of blank)] × 100. IC50 values were obtained from concentration‐response curves, with calculations performed using Excel and GraphPad 9.4.1 software.

### H&E and IHC

Tissues or organoids were harvested, fixed in formalin, and embedded in paraffin for histological sectioning. After dewaxing and rehydration, antigen retrieval was carried out on the tissue sections, which were then treated with 3% hydrogen peroxide for 1 h to block endogenous peroxidase activity. The sections were pre‐incubated with 3% bovine serum albumin for 1 h, followed by overnight incubation at 4 °C with primary antibodies. After applying a horseradish peroxidase‐conjugated secondary antibody, chromogenic staining was performed using DAB (Beyotime), and nuclei were counterstained with hematoxylin.^[^ ^34^ ^]^ The H‐scores were calculated as previously described. Briefly, p63, CK13, PRMT1, PBX2, SMARCC1, and IGF2BP2 were evaluated using a modified histologic score (H‐score), calculated as [{% of weak staining} × 1] + [{% of moderate staining} × 2] + [{% of strong staining} × 3]. In contrast, Ki67 and cleaved caspase‐3 were quantified based on the percentage of positive areas. The results were presented in Figure S14 (Supporting Information).

### Western Blotting

Tissues and cells were lysed using RIPA buffer (Beyotime) to extract total proteins. The proteins were separated by NuPAGE 4–12% Bis‐Tris gels (Invitrogen) and transferred onto a polyvinylidene fluoride (PVDF) membrane (Millipore, MA, USA). The membrane was blocked with 5% skim milk for 1 h, followed by overnight incubation at 4 °C with the primary antibody. Afterward, the membrane was incubated with the appropriate secondary antibody at room temperature for 1 h. Protein bands were visualized using Pierce ECL Western Blotting Substrate (Thermo Fisher Scientific) and detected with a ChemiScope 6100 imaging system (Clinx, Shanghai, China). The Western blot protein bands were analyzed using the Image J software.

### IF Assay

For IF assays, cells were fixed in 4% formaldehyde, permeabilized with 0.5% Triton X‐100 (Sigma‐Aldrich), and blocked with Immunol Staining Blocking Buffer (Beyotime). After overnight incubation with the primary antibody, cells were incubated with fluorophore‐conjugated secondary antibodies for 1 h. Before imaging, cells were mounted with ProLong Gold Antifade Mountant containing DAPI (Thermo Fisher Scientific).

For multiplex immunofluorescent staining of mouse HNSCC tissues, sections were stained with antibodies against PRMT1 and cleaved caspase‐3. The immunocomplexes were visualized using secondary antibodies conjugated with FITC and Cy3, respectively. The sections were counterstained with DAPI (Beyotime) to label nuclei. To quantify cleaved caspase‐3 expression, at least three sections from each HNSCC lesion were immunostained and analyzed. Tumor areas and cleaved caspase‐3 positive areas were manually measured in each section. The percentage of cleaved caspase‐3 positive area was calculated by dividing the cleaved caspase‐3 positive area by the total tumor area. The IF intensity of IGF2BP2 was quantified using ImageJ software, and the values were normalized to the cell count. Results were averaged across the sections.

### Lentivirus Infection

All lentiviral particles were sourced from GenePharma (Shanghai, China), including LV4 lentiviral plasmids expressing PRMT1 cDNA, HA‐PRMT1 cDNA, IGF2BP2 cDNA, HA‐PBX2 cDNA, and an empty control plasmid. The LV‐2 N lentiviral shRNA expression vector was used to target PRMT1, IGF2BP2, SMARCC1, and PBX2, along with a control scrambled shRNA (shSc). Specific target sequences are provided in Table S15 (Supporting Information).^[^ ^35^ ^]^ Lentivirus production and the generation of stable cell lines have been described previously.

### Colony Formation Assay

To evaluate colony formation ability, 1 × 10^3^ cells per well were seeded into 6 cm culture dishes in triplicate. Cells were treated with CBP (45 µM) or DMSO as a control for 48 h. After treatment, the drug‐containing media were removed, and cells were gently washed with PBS before replacing with fresh drug‐free complete culture media. The cells were then allowed to grow undisturbed for 14–16 days to form colonies. At the end of the incubation period, the culture dishes were rinsed with PBS to remove any residual debris and fixed with 4% paraformaldehyde at room temperature for 15 min. Fixed colonies were stained with 0.5% crystal violet solution (Beyotime) for 20–30 min and then gently washed with distilled water to remove excess dye. Colonies containing 50 or more cells were considered significant.

### Cell Apoptosis Analysis

Apoptotic analysis was conducted using an Annexin V‐APC/propidium iodide (PI) Apoptosis Detection Kit (Keygen, Jiangsu, China) according to the manufacturer's instructions. In brief, cells were treated with CBP (45 µM) or DMSO for 24 h, harvested with trypsin (without EDTA), and washed twice with PBS. A staining mixture of 500 µL Binding Buffer, 5 µL Annexin V‐APC, and 5 µL PI was prepared and incubated with the cells in the dark for 10 min. The cells were then analyzed using a flow cytometer (Beckman Coulter, CA, USA).

### Establishment and Culture of HNSCC PDO

PDO cultures were established and maintained according to previously described protocols.^[^ ^34^, ^36^ ^]^ In brief, fresh tumor specimens from HNSCC patients were rinsed three times in ice‐cold PBS, with each rinse lasting 5 min. The tissue was then cut into 1–3 mm^3^ fragments on ice and subjected to enzymatic digestion with trypsin (Sigma‐Aldrich) for ≈30 min. Once the mixture became cloudy, it was filtered through a 100‐µm cell strainer and centrifuged at 200×*g* for 5 min to collect the cell clusters. The clusters were washed three times with PBS for 5 min to remove residual digestive enzymes. After the final wash, the cell clusters were embedded in Matrigel (Corning, NY, USA). Once solidified, the Matrigel‐cell mixture was overlaid with HNSCC organoid medium (BioGenous, Jiangsu, China) and incubated at 37 °C in a CO_2_ incubator, with medium changes every 3 to 5 days.^[^ ^34^ ^]^ Lentiviral transduction was performed as described previously.

For drug testing, viable HNSCC organoids were plated in a 96‐well culture plate at an appropriate density and overlaid with 100 µL of culture medium. After overnight incubation, the medium was replaced with the specified concentrations of saline or CBP. Each experimental group was performed in triplicate. 48 h later, cell viability of the organoids was assessed using the Cell Titer‐Glo 3D cell viability assay (Promega, Madison, WI, USA), following the manufacturer's instructions.

### RNA‐Seq

RNA‐seq was performed by LC Sciences (Hangzhou, China). Total RNA samples underwent thorough quality assessment before being converted into double‐stranded DNA and amplified for 2 × 150 bp paired‐end sequencing (PE150) on an Illumina NovaSeq 6000 platform. Raw sequencing reads were subjected to multiple quality control steps to ensure data quality. Key quality metrics, such as Q20 and Q30 scores, were monitored to confirm that a high proportion of bases met strict quality thresholds. Additionally, mapping rates to the reference genome were evaluated to rule out potential contamination from exogenous sequences. To minimize batch effects, all samples within a given experimental group were processed in the same batch. Furthermore, sample correlation analyses and principal component analysis (PCA) were performed to confirm consistent clustering among biological replicates, ensuring that batch effects did not confound the results. For gene expression quantification, mapped reads were assembled using StringTie with default parameters to compute FPKM values. To address challenges associated with lowly expressed genes and technical noise, DESeq2's normalization algorithm was applied before performing differential expression analysis. Differentially expressed genes (DEGs) were identified using the edgeR R package, with thresholds set at |log₂FC| > 1 and *p*‐value < 0.05 for FaDu cells, and *p*‐value < 0.01 for shPRMT1#1 LIU‐LSC‐1 cells. Multiple testing corrections were applied to control for false positives, ensuring the reliability of the identified DEGs. GO BP over‐representation analysis was performed on the DEGs using the ClusterProfiler R package. The DEG and enrichment pathway results are available in Supplementary Tables S1 and S2 (Supporting Information), and the raw sequencing data have been deposited in NCBI GEO under accession numbers GSE282296 and GSE282312.

### ATAC‐Seq

The ATAC‐seq assay was performed with the assistance of Igenebook (Wuhan, China). Nuclei were extracted from 50 000 cells, washed, and resuspended in nuclear lysis buffer. Transposition was carried out using Tn5 transposase to fragment and tag accessible chromatin regions. Transposed DNA fragments were purified using the Qiagen MinElute PCR Purification Kit (Qiagen, Hilden, Germany), followed by PCR amplification with the addition of unique barcodes. The PCR products were purified to remove excess primers and adapter dimers, and library quality and concentration were assessed using an Agilent Bioanalyzer. High‐throughput sequencing was conducted on the Illumina NovaSeq platform, generating 150 bp paired‐end reads. Raw sequencing data were processed through a standard bioinformatics pipeline, including alignment, peak calling, and differential accessibility analysis. The raw sequencing data have been deposited in the NCBI GEO database under accession number GSE282295. Differentially accessible regions (DARs) were identified and annotated, with results presented in Table S3 (Supporting Information).

### Plasmids Transfection

The FL PRMT1 plasmids tagged with Flag (amino acids 1–353), truncated PRMT1 plasmids with Flag (PRMT1‐fragment 1: amino acids 1–22, 177–353; PRMT1‐fragment 2: amino acids 1–175), and mutated PRMT1 plasmids (A26K, S31A, K32A, H41A, Y107A, and G80R) were provided by Tsingke Biotech (Beijing, China). The mutated PRMT1 plasmids with Flag (M48A, M155A) were provided by GENEWIZ (Suzhou, China). Additionally, HA‐tagged SMARCC1 plasmids (amino acids 1–1105) and their truncated versions (SMARCC1‐fragment 1: amino acids 1–616; SMARCC1‐fragment 2: amino acids 617–908; SMARCC1‐fragment 3: amino acids 909–1105), as well as mutated SMARCC1 plasmids (K34A, D35A, K94A, D215A, and Y236A), were also provided by Tsingke Biotech. The shRNA‐resistant PRMT1 plasmid was obtained from GenePharma. Plasmid transfection was carried out using Lipofectamine 3000 reagents (Thermo Fisher Scientific) according to the manufacturer's instructions.

### Mass Spectrometry

LIU‐LSC‐1 cell lysates were subjected to immunoprecipitation. Followed by centrifugation to collect the supernatant. PRMT1 or IgG antibodies were incubated with magnetic beads at room temperature for 1 h. The supernatant was then added to the magnetic beads and incubated overnight at 4 °C. After elution, the samples were separated using a 4–12% SDS‐PAGE gel, and the gel bands were excised and collected. Protein interactions with PRMT1 were analyzed by liquid chromatography‐mass spectrometry (LC‐MS), which was performed by Bioprofile (Shanghai, China). The raw IP‐MS data was updated to iProX database (IPX0011391000).

### GST Pull‐Down Assay

The human PRMT1 cDNA was cloned into the pGEX‐6p‐2 GST fusion vector (Cytiva, MA, USA), and the resulting GST‐tagged plasmids were transformed into Escherichia coli BL21 strains. Protein expression in the transformed BL21 strains was induced at 16 °C for 12 h to produce GST fusion proteins. The recombinant GST‐tagged proteins were purified using glutathione‐Sepharose resin (Cytiva, MA, USA). For the pull‐down assay, equal amounts of GST‐PRMT1 protein and GST control protein (used as a negative control) were conjugated to glutathione‐agarose beads (Cytiva) and incubated with lysates from HEK293T cells transfected with HA‐SMARCC1 constructs (Tsingke Biotech) at 4 °C for 3 h. Following incubation, the GST pull‐down complexes were washed five times with NETN buffer, resuspended in 2× loading buffer, boiled for 5 min, and subsequently analyzed by immunoblotting.

### Co‐IP Assay

After different treatments, HNSCC cells were washed with cold PBS and lysed in cell lysis buffer (Beyotime) on ice. The total protein content in the lysate was designated as the “Input” For IP, PRMT1, SMARCC1, HA, Flag, or IgG antibodies were incubated with 20 µL of protein G beads at room temperature for 1 h. The antibody‐bead mixture was then added to the cell lysate and incubated overnight at 4 °C. Following incubation, the beads were washed three times with IP washing buffer. After washing, 20 µL of 2× SDS loading buffer (Beyotime) was added, and the mixture was heated at 95 °C for 5 min. Western blotting analysis was performed using antibodies specific to the indicated proteins to assess protein‐protein interactions. Specific secondary antibodies were used to avoid interference from heavy chains.

### Molecular Docking

Molecular docking was performed using the HDOCK online platform (http://hdock.phys.hust.edu.cn/), which evaluates binding affinities across various conformations. The 3D structures of the SMARCC1 and PRMT1 proteins were modeled using the SWISS‐MODEL server. Docking simulations were conducted via HDOCK to predict interactions between SMARCC1 and PRMT1. The resulting interactions were visualized and analyzed in both 3D and 2D formats using PyMOL and LigPlot+ software.

### CUT&Tag Assays

The CUT&Tag analysis was conducted in collaboration with Igenebook (Wuhan, China). Cells were prepared, counted, and washed to remove debris, followed by sequential incubation with a primary antibody specific to the target protein and a secondary antibody conjugated to protein A or G to facilitate ChiTag transposase (Vazyme, Nanjing, China) binding. After antibody binding, the cells were treated with ChiTag transposase for DNA fragmentation and tagging of accessible chromatin regions. The tagged DNA fragments were purified using magnetic beads and amplified by PCR with indexed primers for sample multiplexing. The final PCR products were purified, and library quality, including concentration and fragment size, was assessed using a Qubit fluorometer and capillary electrophoresis. Libraries meeting quality standards were denatured, diluted, and sequenced on the Illumina NovaSeq platform following cluster generation. Sequencing data were analyzed using standard bioinformatics workflows, including alignment, peak calling, and differential analysis of transcription factor binding sites. The raw sequencing data have been deposited in the NCBI GEO database under accession number GSE282361, providing full access to the analyzed results. Differential binding peaks (DBPs) are listed in Table S7 (Supporting Information).

### Reporter Constructs and Luciferase Reporter Assay

A 579 bp segment of the human PRMT1 gene (−426/+153), which includes the PBX2‐binding region, was amplified from human genomic DNA by PCR. The resulting PCR product was inserted into the luciferase reporter gene vector pGL3‐Basic (Promega) using *Kpn* I and *Bgl* II restriction sites. The primer sequences used for PCR amplification were as follows: forward, 5′‐GGGGTACCCCGGATAAACCAATGGCAG‐3′; reverse, 5′‐GGAAGATCTGGACCTCCAACCCCATATC‐3′. To generate a mutated version of this construct, designated pPRMT1mut‐Luc, the PBX2‐binding site within the amplified region was altered using the Q5 Site‐Directed Mutagenesis Kit (NEB, MA, USA). The mutation primers used were: forward, 5′‐GTTTTGGTGAATACCAGGTGTTTCAAGACGCCC‐3′; reverse, 5′‐GACCCGCCTCCTAATCGC‐3′. HEK293T cells were seeded in 24‐well plates and co‐transfected with 200 ng of either the PRMT1wt‐Luc or pPRMT1mut‐Luc plasmids, along with 200 ng of either pcDNA3.1‐PBX2 or control vector pcDNA3.1, and 10 ng of the internal control plasmid pRL‐TK. Luciferase activity was measured using the Dual‐Luciferase Reporter Assay System (Promega), and the relative luciferase activity was normalized to the Renilla luciferase internal control.

### ChIP & ChIP‐reChIP

ChIP assay was performed using the SimpleChIP Plus Enzymatic Chromatin IP Kit (Cell Signaling Technology) according to the manufacturer's instructions. Briefly, cells were cross‐linked with 1% formaldehyde for 10 min, lysed with sodium dodecyl sulfate (SDS) lysis buffer, and subjected to ultrasonication for 30 min. The chromatin was then incubated overnight with antibodies against HA or SMARCC1. ChIP‐reChIP assays were performed by Re‐ChIP‐IT kit (Active Motif, Carlsbad, CA) following the manufacturer's instructions. Control rabbit IgG or anti‐HA antibody for the first ChIP and control rabbit IgG or anti‐SMARCC1 antibody was used for the reChIP. The immunoprecipitated DNA was subsequently purified and analyzed by PCR or qPCR using specific primers, which are listed in Table S16 (Supporting Information).

### In Vivo Tumor Models

Healthy male BALB/c nude mice and NOD/SCID were obtained from Gempharmatech (Jiangsu, China). The animals were quarantined and housed at the Animal Experiment Center of Anhui Medical University, where they were cared for according to protocols approved by the university's Ethics Committee.

For CDX assays, six‐week‐old BALB/c nude mice were randomly assigned to groups of five and treated with various regimens. A total of 4 × 10^6^ cells, suspended in 0.2 mL of PBS, were injected subcutaneously into the axillary region to establish tumor models. On the fifteenth day after cell inoculation, the mice were treated with either CBP (25 mg kg^−1^, twice weekly) or saline.

The HNSCC PDX models were established as previously described.^[^ ^33^ ^]^ When the third‐passage tumors reached an average volume of 100 mm^3^, the mice were randomly assigned to four groups (*n* = 5 per group) and treated with either PRMT1 siRNA (100 µg, three times weekly) or a non‐targeting siRNA control (siNC), in combination with CBP (25 mg kg^−1^, twice weekly) or saline.

CBP was administered via intraperitoneal injection, while chemically modified PRMT1 siRNAs (GenePharma) were delivered intratumorally. Tumor size was measured every three days using a digital caliper, and volumes were calculated using the formula: Volume (mm^3^) = 0.5 × (Length × Width^2^). Body weights were monitored throughout the experiment. At the study endpoint, as indicated in the respective figures, animals were euthanized, and subcutaneous tumor masses were excised for further analysis.

### Conditional Knockout Mice Model

The *Prmt1^fl/fl^* and *Krt14^Cre/ERT2^* mice strains were interbred to generate *Prmt1^fl/fl^; Krt14^Cre/ERT2^* mice, which were obtained from the Shanghai Model Organisms Center and maintained under specific pathogen‐free (SPF) conditions. To induce HNSCC, six‐week‐old mice *Prmt1^wt/wt^; Krt14^Cre/ERT2^* (*Prmt1^Ctrl^*) and *Prmt1^fl/fl^*; *Krt14^Cre/ERT2^* (*Prmt1^cKO^*) were provided drinking water containing 50 µg mL^−1^ 4NQO for 16 weeks, after which they were switched to regular drinking water to facilitate tumor development. For lineage tracing and PRMT1 knockout experiments, tamoxifen was administered to mice through intraperitoneal injections (120 mg kg^−1^, every other day) for 4 times to activate Cre recombinase. In addition, the mice were given intraperitoneal injections of CBP (25 mg kg^−1^) or saline twice weekly for a duration of four weeks. After completing the treatment protocol, the mice were euthanized, and tongue tissue samples were collected for subsequent analysis.

### Bioinformatics Analysis

The single‐cell RNA‐seq dataset GSE181919 was sourced from the GEO. It includes data from 24 HNSCC and 9 ANM tissues. Epithelial and malignant cells were specifically isolated from each sample based on the accompanying metadata for further analysis. Library size normalization for each individual cell was carried out using the NormalizeData function in Seurat (version 4.1.1).^[^ ^37^ ^]^ For data visualization and plotting, ggplot was employed, and comparisons between groups were performed using the stat_compare_means function.

Clinical information for HNSCC was retrieved from The Cancer Genome Atlas (TCGA) database, available at http://cancergenome.nih.gov/. The diagnostic potential of PBX2, PRMT1, IGF2BP2, and SMARCC1 was evaluated through Receiver Operating Characteristic ROC curve analysis to assess sensitivity and specificity. For the CBP treatment subgroup in TCGA‐HNSCC, multivariate Cox regression analysis was performed to evaluate the prognostic impact of gene expression, adjusting for clinical variables such as age, gender, clinical tumor stage, T‐stage, and M‐stage. Kaplan‐Meier survival analysis was generated by stratifying patients into high‐ and low‐expression groups based on optimal cutoff values derived from gene expression levels, followed by survival analysis. The log‐rank test was used to calculate the statistical significance of differences in survival between the two groups. Based on the full gene mRNA expression, The OncoPredict R package was used to predict CBP sensitivity for each sample. DEGs between these CBP‐sensitive samples (top 25%) and CBP‐resistant samples (bottom 25%) were identified using the Wilcoxon test, enabling the detection of genes associated with differences in carboplatin sensitivity.

### Statistical Analysis

Data are expressed as mean ± standard deviation (SD). GraphPad Prism 9.4.1 software was used to calculate the *p*‐value using Student's *t*‐test for between‐group comparisons and one‐way ANOVA or two‐way ANOVA for multiple comparisons. *p*‐value of <0.05 was considered statistically significant (*p < 0.05, **p < 0.01, ***p < 0.001, ****p < 0.0001).

## Conflict of Interest

The authors declare no conflict of interest.

## Author Contributions

S.L., W.Z., W.L., and Z.D. contributed equally to this work. X.J.Z., S.Y.M., and Y.H.L. supervised the project; X.J.Z., S.Y.M., and Y.H.L. designed the experiments; S.X.L., W.T.Z., W.W.L., Z.D., Z.X.W., R.J.Z., Y.W., Y.X.H., Z.H.X., and J.W. performed the experiments; S.X.L., W.T.Z., Y.F.H., H.L., D.P.L., J.P., Z.H.N., M.D.Z., W.J., Y.L.H., L.Q., and Z.D.H. analyzed the data; X.J.Z. and S.X.L. wrote the paper; X.J.Z., S.Y.M., Y.H.L., and X.C. revised the paper. All authors read and approved the manuscript.

## Supporting information



Supporting Information

Supplemental Table 1

Supplemental Table 2

Supplemental Table 3

Supplemental Table 4

Supplemental Table 5

Supplemental Table 6

Supplemental Table 7

## Data Availability

All data generated and analyzed during this study are included in this published article and supplementary information. Materials generated during the present study are available from the corresponding author on reasonable request.
