# Supplementary material for: PRMT1‐Mediated SWI/SNF Complex Recruitment via SMARCC1 Drives IGF2BP2 Transcription to Enhance Carboplatin Resistance in Head and Neck Squamous Cell Carcinoma
Source: Adv Sci (Weinh). 2025 Apr 24;12(22):2417460. doi: 10.1002/advs.202417460 (PMC12165065; doi:10.1002/advs.202417460)
Supplement: Supplementary file 1 — Supporting Information [file ADVS-12-2417460-s005.docx]

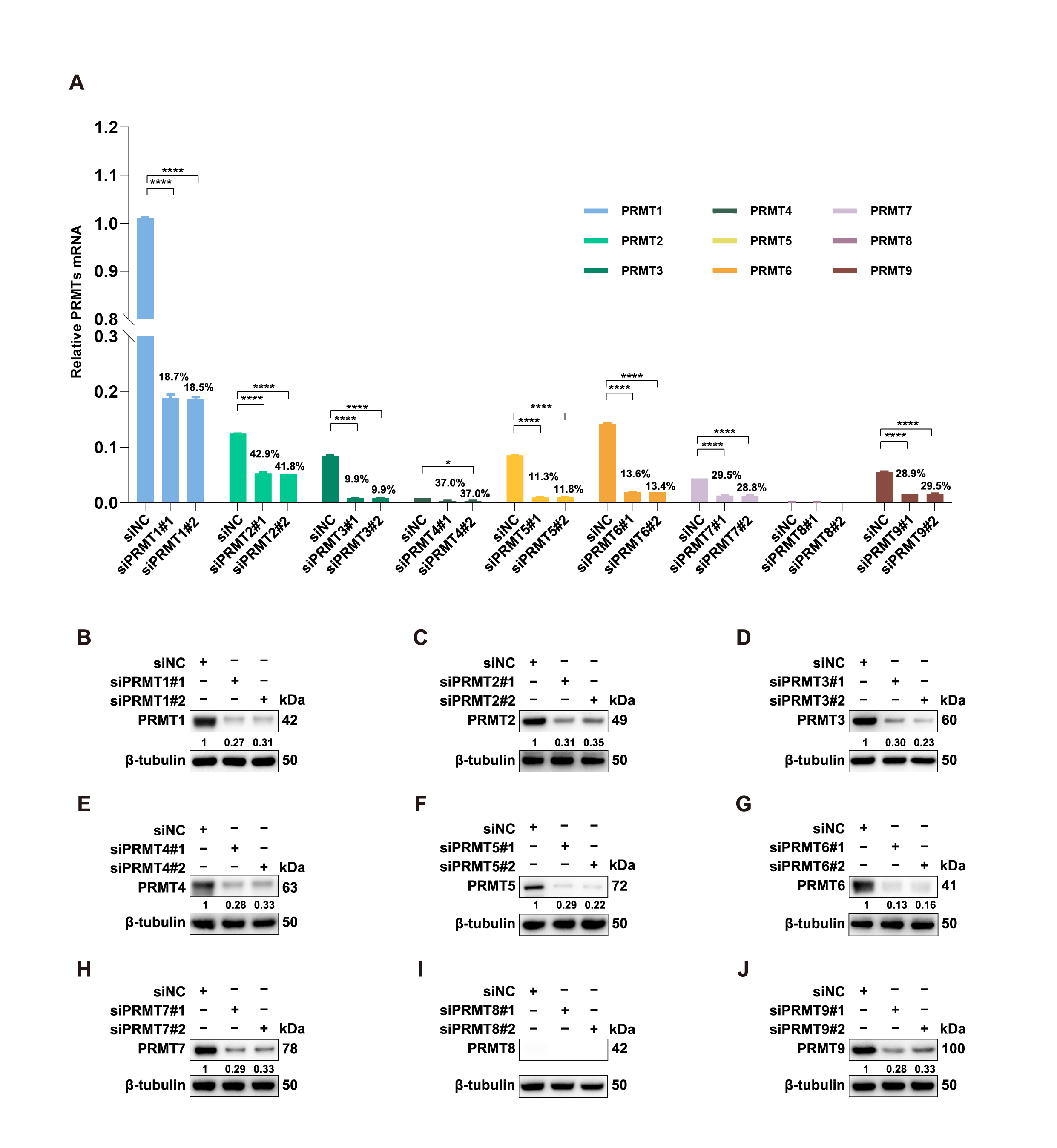


**Figure S1 Verification of siRNA-mediated knockdown efficiency for PRMT family genes in FaDu cells.**

FaDu cells were transfected with siRNAs targeting PRMTs or a scrambled sequence (siNC) for 48 h. The expression levels of PRMTs were assessed using qRT-PCR (A) and western blotting (B-J). Error bars represent mean ± SD; *P < 0.05, ****P < 0.0001.


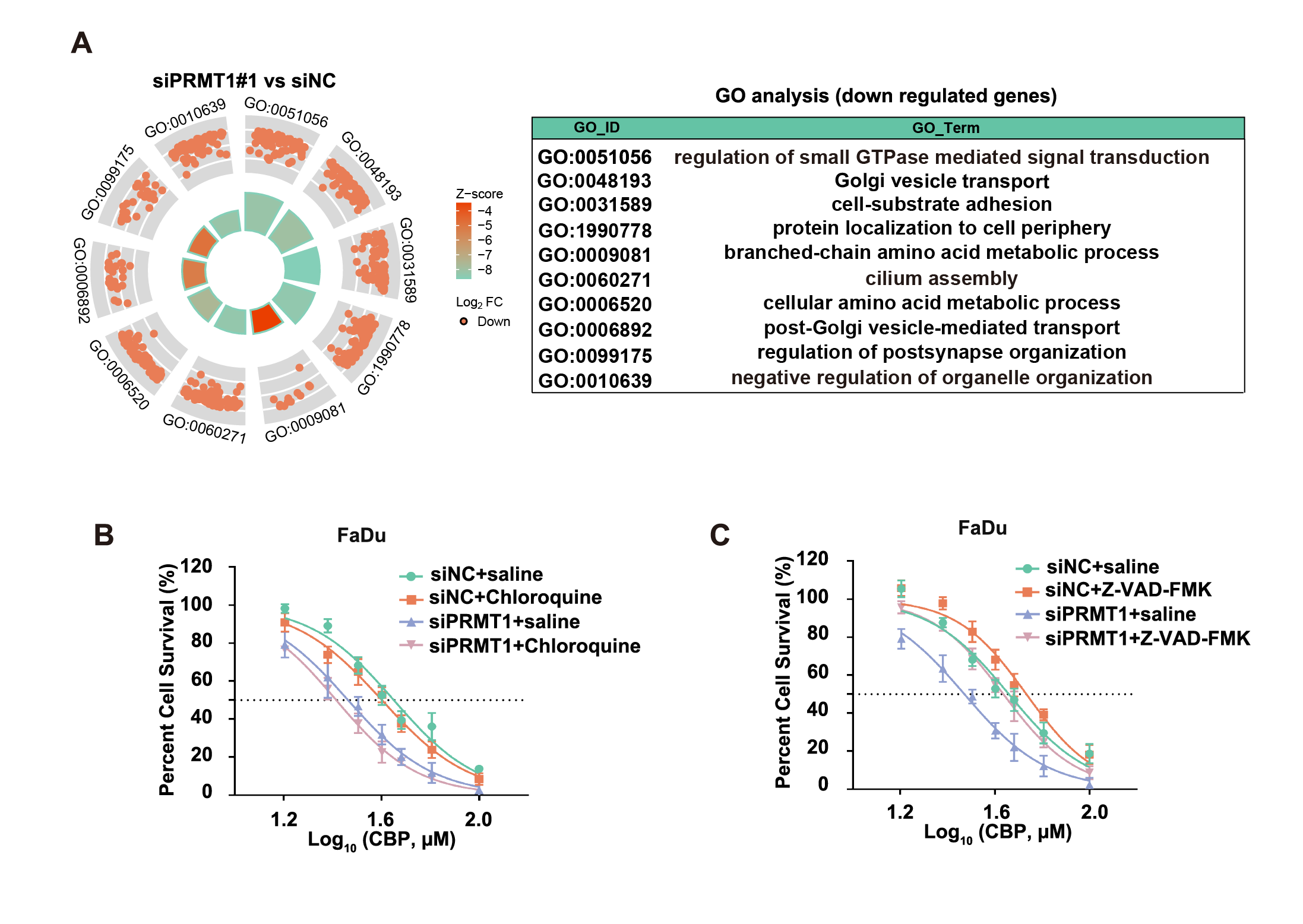


**Figure S2 PRMT1 confers resistance to CBP by suppressing apoptosis.**

A) The top 10 GO BP pathways were enriched among the downregulated DEGs identified by RNA-seq in PRMT1 knockdown (siPRMT1#1) and control (siNC) FaDu cells following CBP treatment (45 μM, 72 h). B, C) FaDu cells transfected with siPRMT1#1 or siNC were pretreated with either 50 µM chloroquine (autophagy inhibitor, B), 40 µM Z-VAD-FMK (apoptosis inhibitor, C), or saline for 1 h, followed by CBP treatment (45 µM) for 48 h. The IC50 of CBP was then determined to assess its cytotoxicity under each condition. Error bars represent mean ± SD.


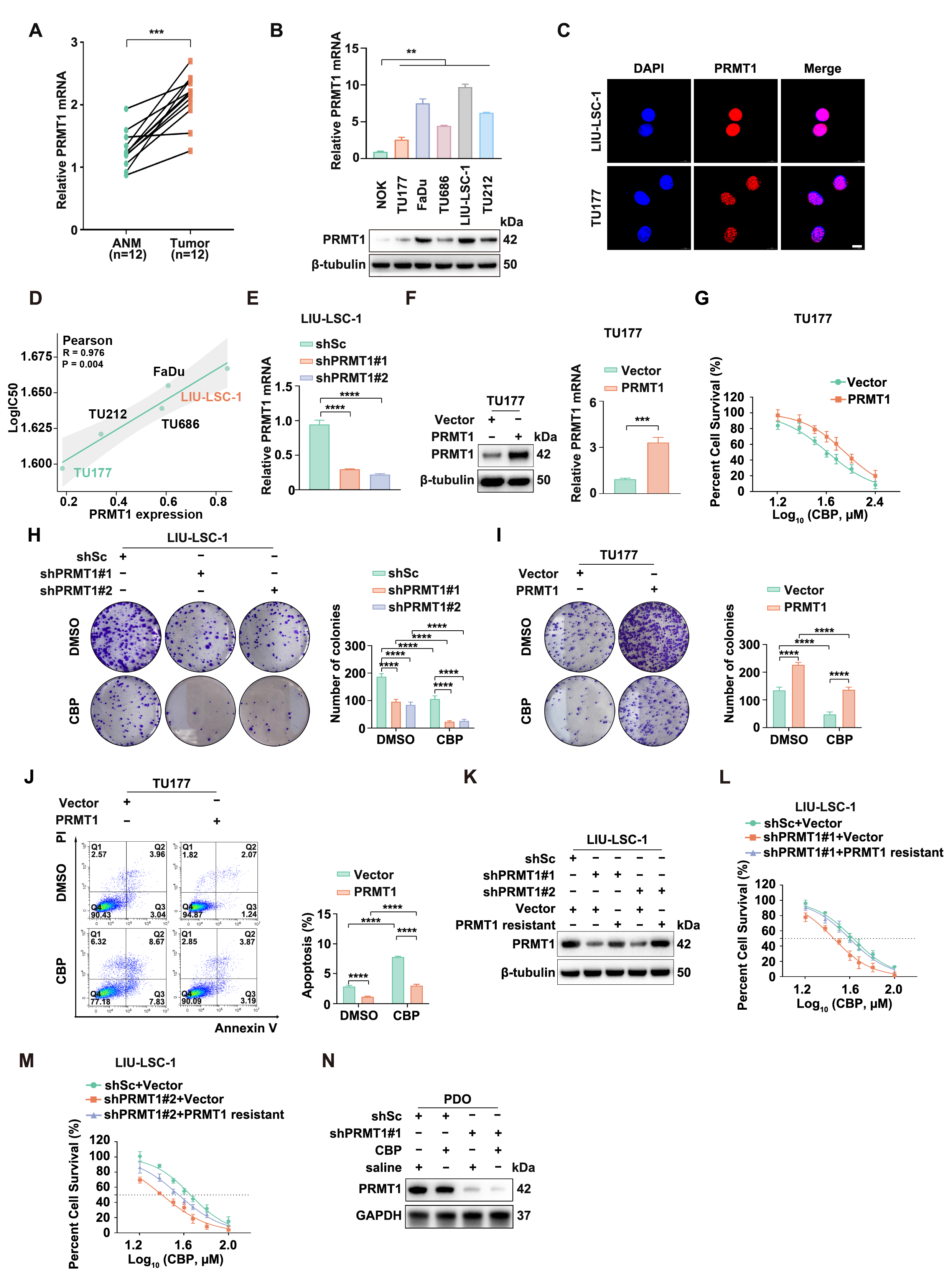


**Figure S3 PRMT1 enhances the resistance of HNSCC cells to CBP.**

A) qRT-PCR was performed to evaluate mRNA levels of PRMT1 in paired ANM and tumor tissues (n = 12). Error bars represent mean ± SD; ***P < 0.001. B) The expression levels of PRMT1 in NOK, TU177, FaDu, TU686, LIU-LSC-1, and TU212 cell lines were assessed using qRT-PCR and western blotting analysis. Error bars represent mean ± SD; **P < 0.01. C) Representative immunofluorescence images depicting PRMT1 expression in LIU-LSC-1 and TU177 cells are presented. Scale bar: 10 μm. D) The Pearson's correlation between PRMT1 expression levels and CBP logIC50 values in five HNSCC cell lines is shown, with the correlation coefficient and p-value displayed in the upper left corner. A fitted trend line represents the linear regression, and the shaded area depicts the 95% confidence interval. The names of each cell line are labeled next to the corresponding data points. E, H) LIU-LSC-1 cells were infected with lentiviruses expressing shRNAs targeting PRMT1 (shPRMT1#1 and shPRMT1#2) or a control scrambled sequence (shSc). F, G, I, J) Lentiviruses encoding PRMT1 cDNA or a control vector were used to infect TU177 cells. E, F) PRMT1 expression in the indicated cells were detected by qRT-PCR and western blotting. Error bars represent mean ± SD; ***P < 0.001, ****P < 0.0001. G, L, M) The CCK-8 assay was utilized to determine the IC50 values of CBP. H, I) Cells were pre-treated with CBP (45 µM, 48 h) or DMSO, and cell growth ability was assessed using colony formation assays. Error bars represent mean ± SD; ***P < 0.001, ****P < 0.0001. J) Cells were treated with CBP (45 µM, 24 h) or DMSO, and cell apoptosis was measured by flow cytometry. Error bars represent mean ± SD; ****P < 0.0001. K-M) LIU-LSC-1 cells with PRMT1 knockdown (shPRMT1#1 and shPRMT1#2) were transfected with shRNA-resistant PRMT1 constructs or a control Vector. N) shPRMT1#1 or shSc-expressing HNSCC organoids were treated with CBP (45 µM) or saline for 48 h. PRMT1 levels were then detected by western blotting analysis.


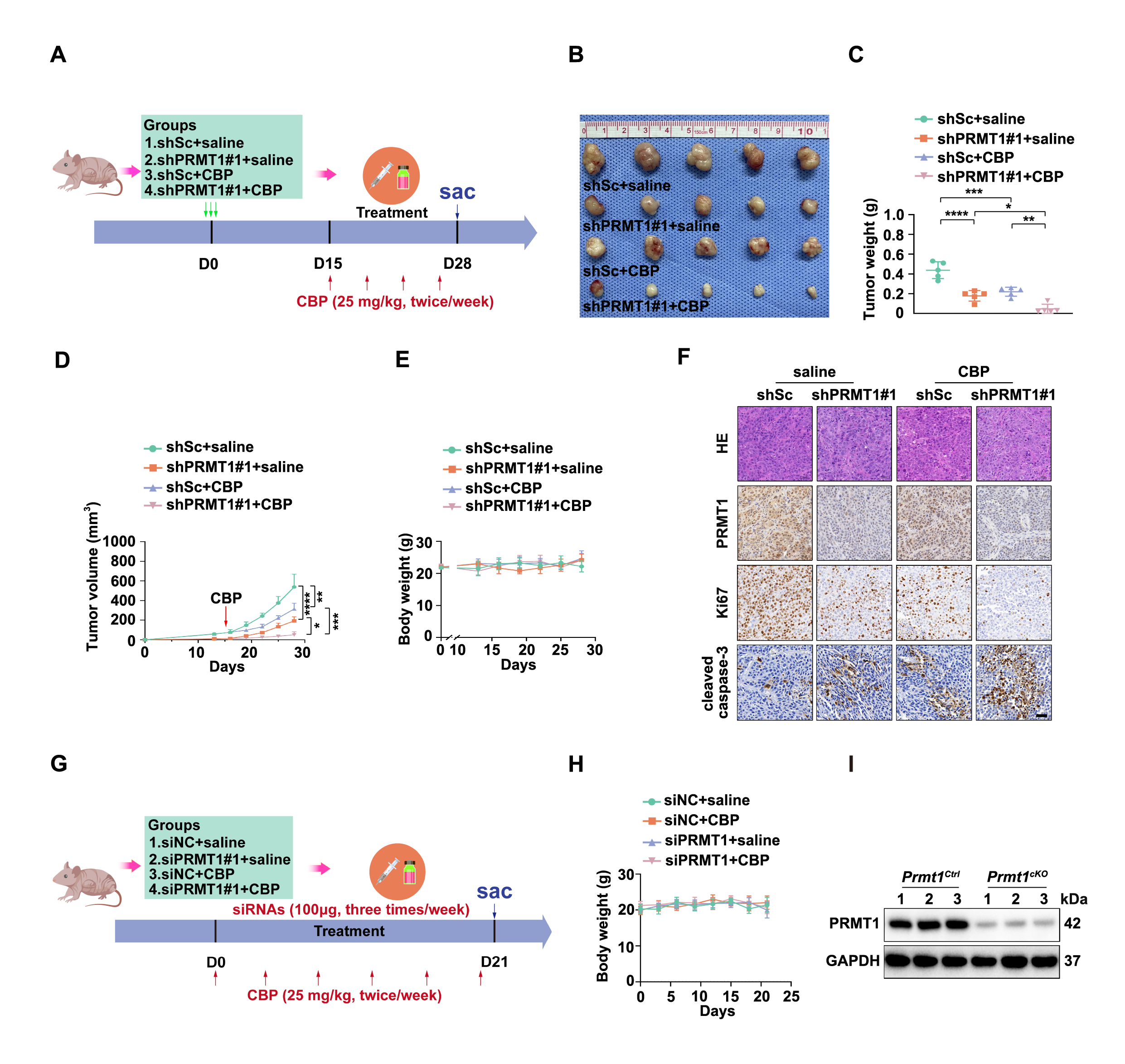


**Figure S4 Depletion of PRMT1 enhances the *in vivo* anti-tumor efficacy of CBP.**

A-F) The indicated cells were injected subcutaneously into immunodeficient mice, followed by CBP (25 mg/kg, i.p., twice/week) or saline treatment, and tumor growth was monitored. A diagrammatic representation of the experimental framework and timeline is shown (A). Tumor images (B), tumor weights (C), tumor volumes (D), body weight (E), and representative IHC staining of PRMT1, Ki67, and cleaved caspase-3 in xenograft tissues are presented (F). Error bars represent mean ± SD; *P < 0.05, **P < 0.01, ***P < 0.001, ****P < 0.0001. Scale bar: 50 μm. G) A diagrammatic representation of the experimental design and timeline for HNSCC PDX tumor models treated with PRMT1 siRNA, with or without CBP, is shown. H) Changes in body weight of mice bearing PDX tumor models following treatment with PRMT1 siRNAs or siNC, in combination with CBP (25 mg/kg, i.p., twice/week) or saline, are shown (n = 5 mice per group). I) Western blotting analysis was employed to evaluate PRMT1 protein expression levels in the tongue lesions from *Prmt1^Ctrl^* and *Prmt1^cKO^* mice (n = 3).


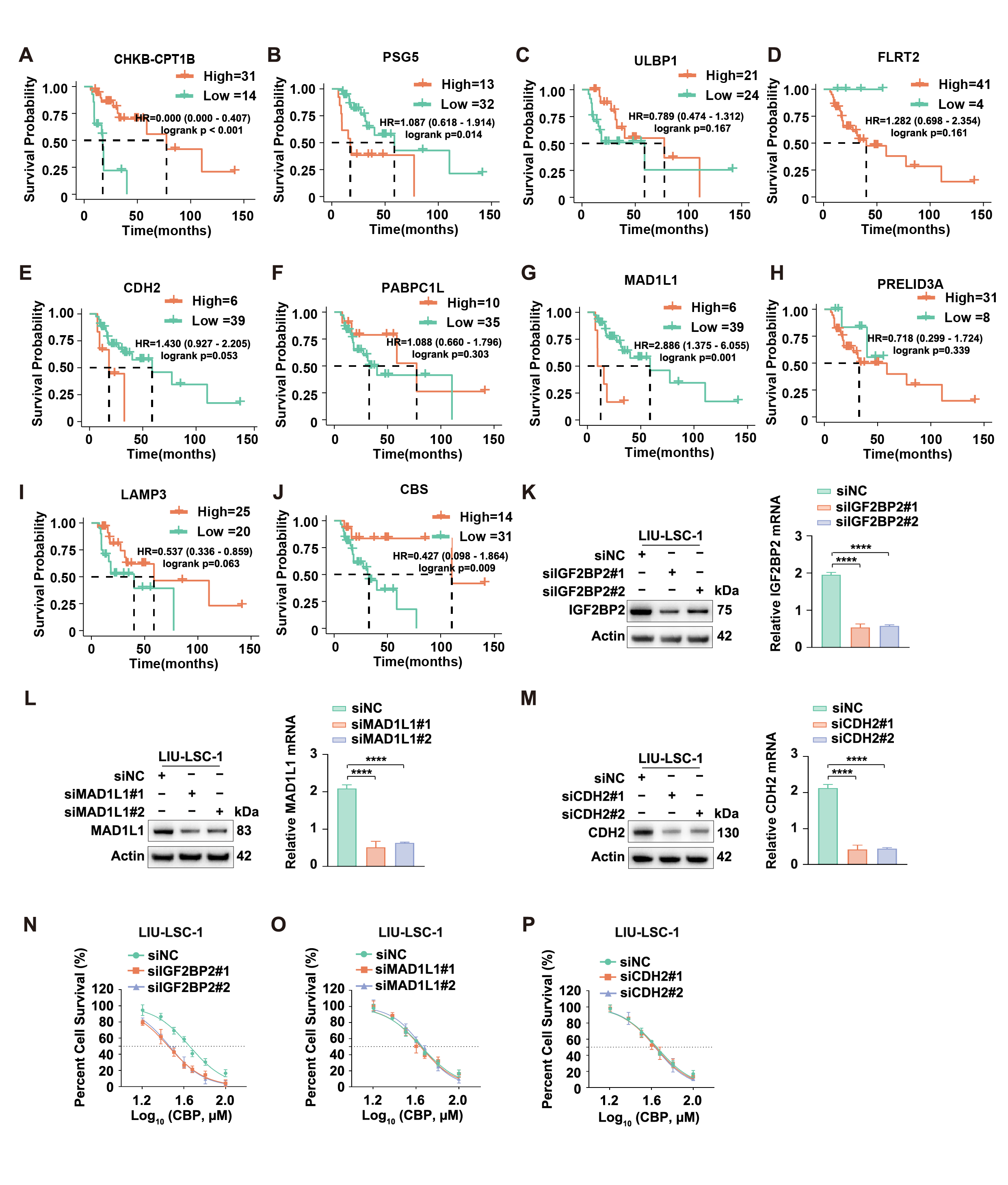


**Figure S5 Correlation between IGF2BP2 expression and CBP sensitivity in HNSCC.**

A-J) Kaplan-Meier survival analysis was conducted for HNSCC patients in the TCGA-HNSCC subgroup, which accepted CBP treatment (n = 45). Patients were stratified into high and low expression groups based on the optimal cutoff values for the expression levels of CHKB-CPT1B, PSG5, ULBP1, FLRT2, CDH2, PABPC1L, MAD1L1, PRELID3A, LAMP3, and CBS. K-P) LIU-LSC-1 cells were transfected with siRNAs targeting IGF2BP2, MAD11L and CDH2, or with siNC for 48 h. The expression levels of IGF2BP2 (K), MAD11L (L) and CDH2 (M) were assessed using western blotting (left panels) and qRT-PCR (right panels). The CCK-8 assay was utilized to determine the IC50 values of CBP (N-P). Error bars represent mean ± SD. n.s.: not significant; ***P < 0.001.


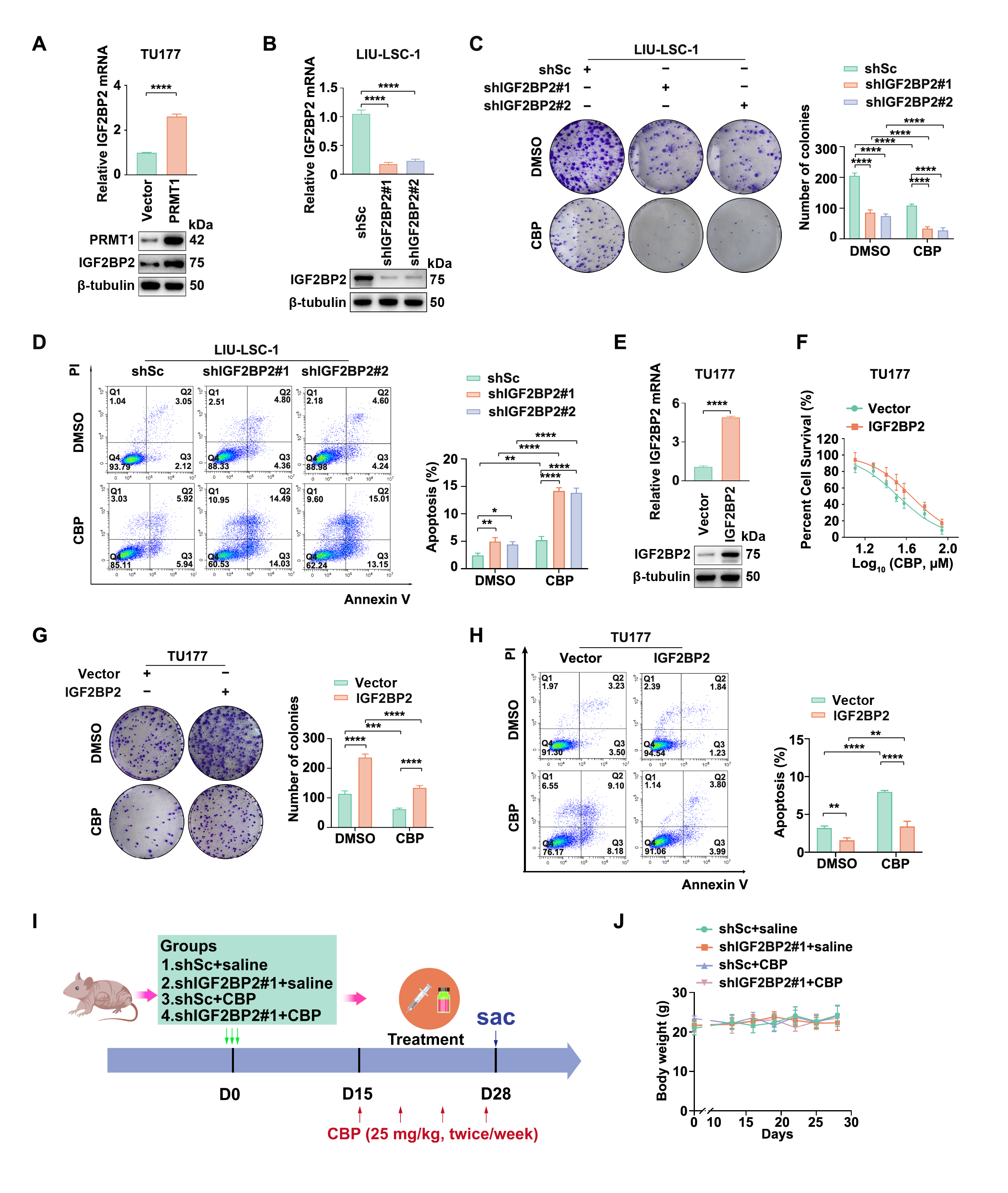


**Fig.S6 IGF2BP2 enhances the resistance of HNSCC cells to CBP.**

A) The levels of IGF2BP2 in PRMT1-overexpressing TU177 cells and control cells were assessed using qRT-PCR and western blotting analysis. Error bars represent mean ± SD. ****P < 0.0001. B-D) LIU-LSC-1 cells were transduced with lentiviral shIGF2BP2#1, shIGF2BP2#2, or a scrambled control (shSc). The expression of IGF2BP2 were evaluated by qRT-PCR and western blotting (B). Cells were pre-treated with CBP (45 µM, 48 h) or DMSO, and cell growth ability was assessed using colony formation assays (C). Cells were treated with CBP (45 µM, 24 h) or DMSO, and apoptosis was measured by flow cytometry (D). Error bars represent mean ± SD. *P < 0.05, **P < 0.01, ****P < 0.0001. E-H) TU177 cells were infected with lentiviruses overexpressing IGF2BP2 or a control vector. The expression of IGF2BP2 were evaluated by qRT-PCR and western blotting (E). The CCK-8 assay was performed to determine the IC50 values of CBP (F). Cells were pre-treated with CBP (45 µM, 48 h) or DMSO, and cell growth ability was assessed using colony formation assays (G). Cells were treated with CBP (45 µM, 24 h) or DMSO, and apoptosis was measured by flow cytometry (H). Error bars represent mean ± SD. **P < 0.01, ***P < 0.001, ****P < 0.0001. I) A diagrammatic representation of the experimental design and timeline for CDX tumors derived from shIGF2BP2#1-transduced LIU-LSC-1 cells and control cells treated with either CBP (25 mg/kg, i.p., twice/week) or saline is presented. J) Changes in body weight of mice bearing CDX tumors following treatment with CBP (25 mg/kg, i.p., twice/week) or saline. n = 5 mice per group.


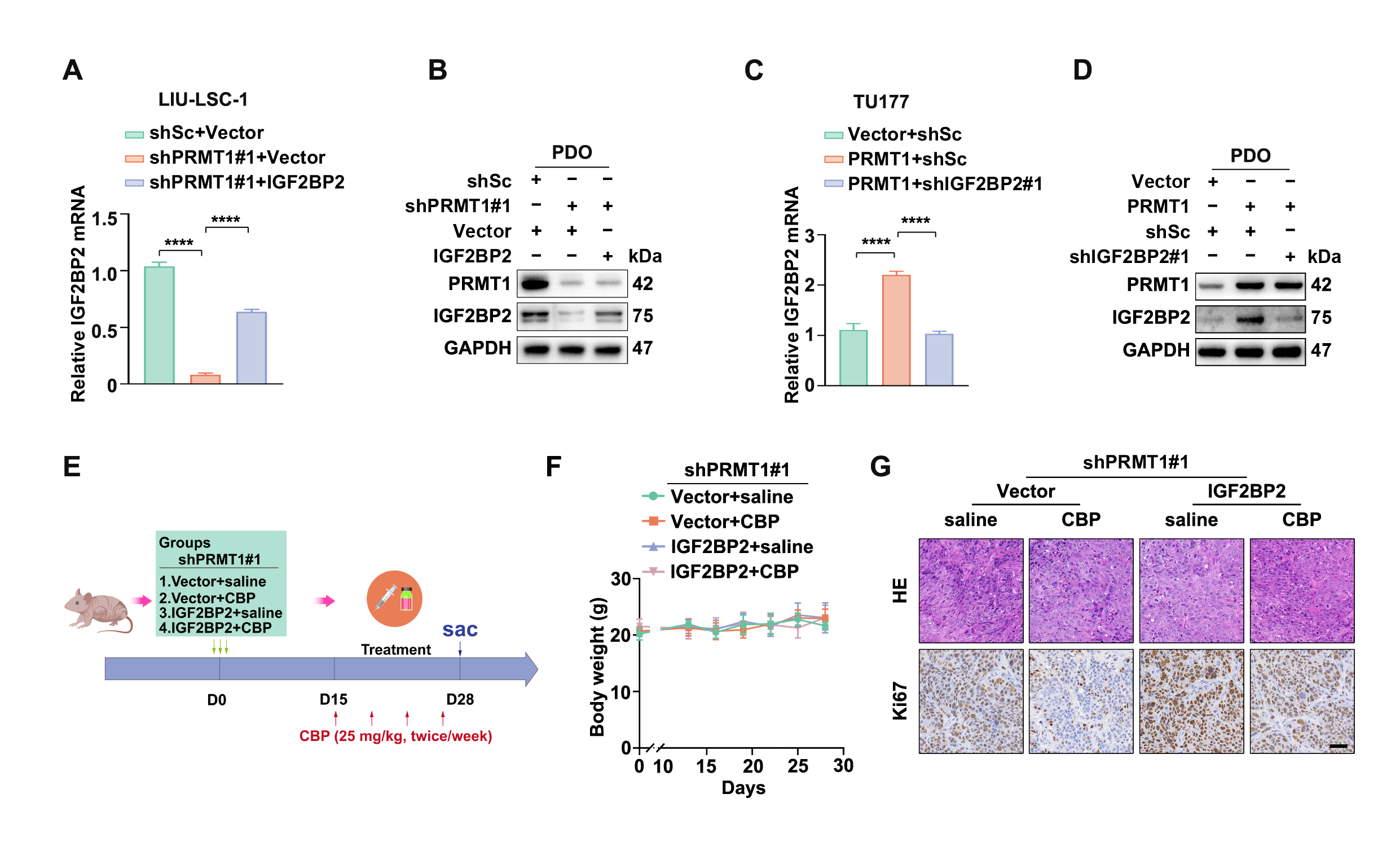


**Figure S7 PRMT1 promotes resistance to CBP in HNSCC cells through the upregulation** **of IGF2BP2.**

A) shPRMT1#1 LIU-LSC-1 cells and control cells were transduced with lentiviruses containing either a vector encoding human *IGF2BP2* cDNA or an empty control vector. The expression of IGF2BP2 was assessed using qRT-PCR. Error bars represent mean ± SD. ****P < 0.0001. B) HNSCC organoids with PRMT1 knockdown were transduced with lentiviruses carrying either a vector encoding human *IGF2BP2* cDNA or an empty control vector. Western blotting analysis was performed to evaluate the protein expression levels of PRMT1 and IGF2BP2. C) Lentiviruses expressing shIGF2BP2#1 or shSc were introduced into PRMT1-overexpressing TU177 cells and control cells. The expression of IGF2BP2 was assessed using qRT-PCR. Error bars represent mean ± SD. ****P < 0.0001. D) PRMT1-overexpressing HNSCC organoids and control organoids were transduced with lentiviruses expressing shIGF2BP2#1 or shSc. Western blotting analysis was performed to evaluate the protein expression levels of PRMT1 and IGF2BP2. E) A diagrammatic representation of the experimental design and timeline for CDX tumors derived from the indicated cells treated with either CBP (25 mg/kg, i.p., twice/week) or saline is shown. F) Changes in body weight of mice bearing CDX tumors following treatment with CBP (25 mg/kg, i.p., twice/week) or saline are depicted, with n = 5 mice per group. G) Representative IHC images illustrating Ki67 expression in xenograft tissues are provided. Scale bar: 50 μm.


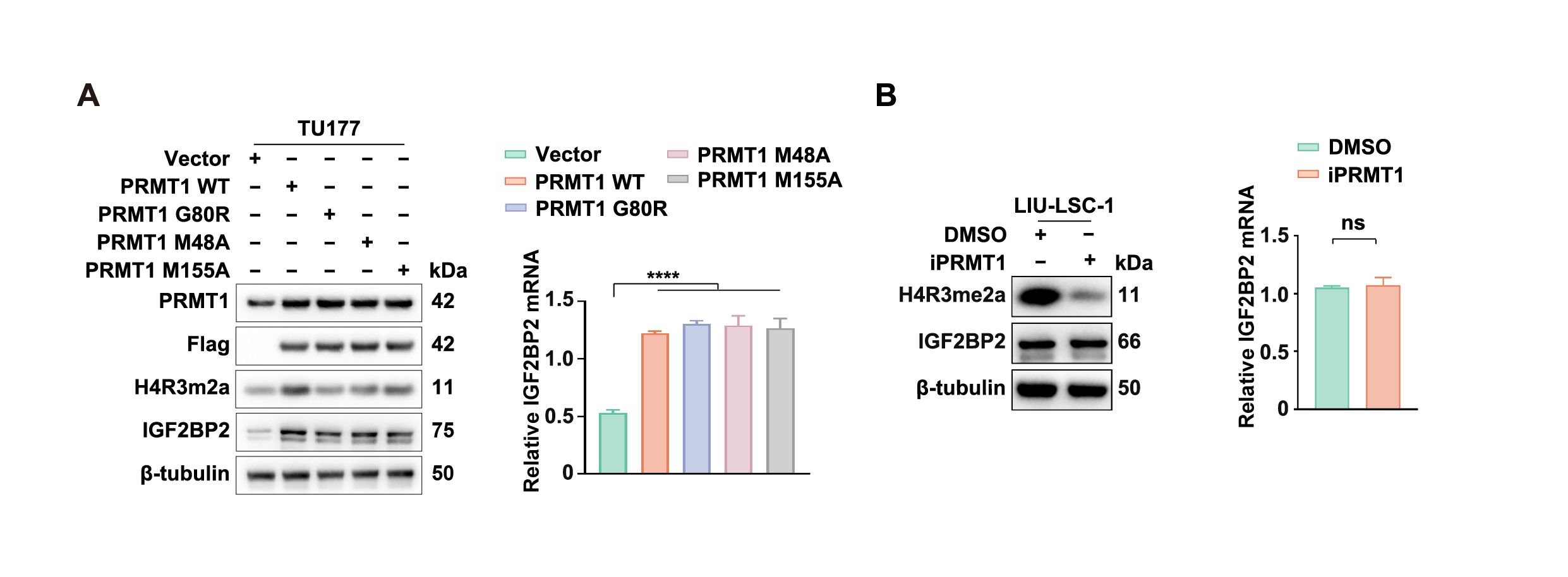


**Figure S8 PRMT1 upregulates IGF2BP2 independently of its enzymatic function.**

A) TU177 cells were transfected with wild-type PRMT1 and mutant constructs, including PRMT1 G80R, PRMT1 M48A, and PRMT1 M155A. B) LIU-LSC-1 cells were treated with either iPRMT1 (0.1 µM) or DMSO for 24 h. The expression levels of IGF2BP2 were assessed using western blotting (left panels) and qRT-PCR (right panels). Error bars represent mean ± SD. n.s.: not significant; ****P < 0.0001.


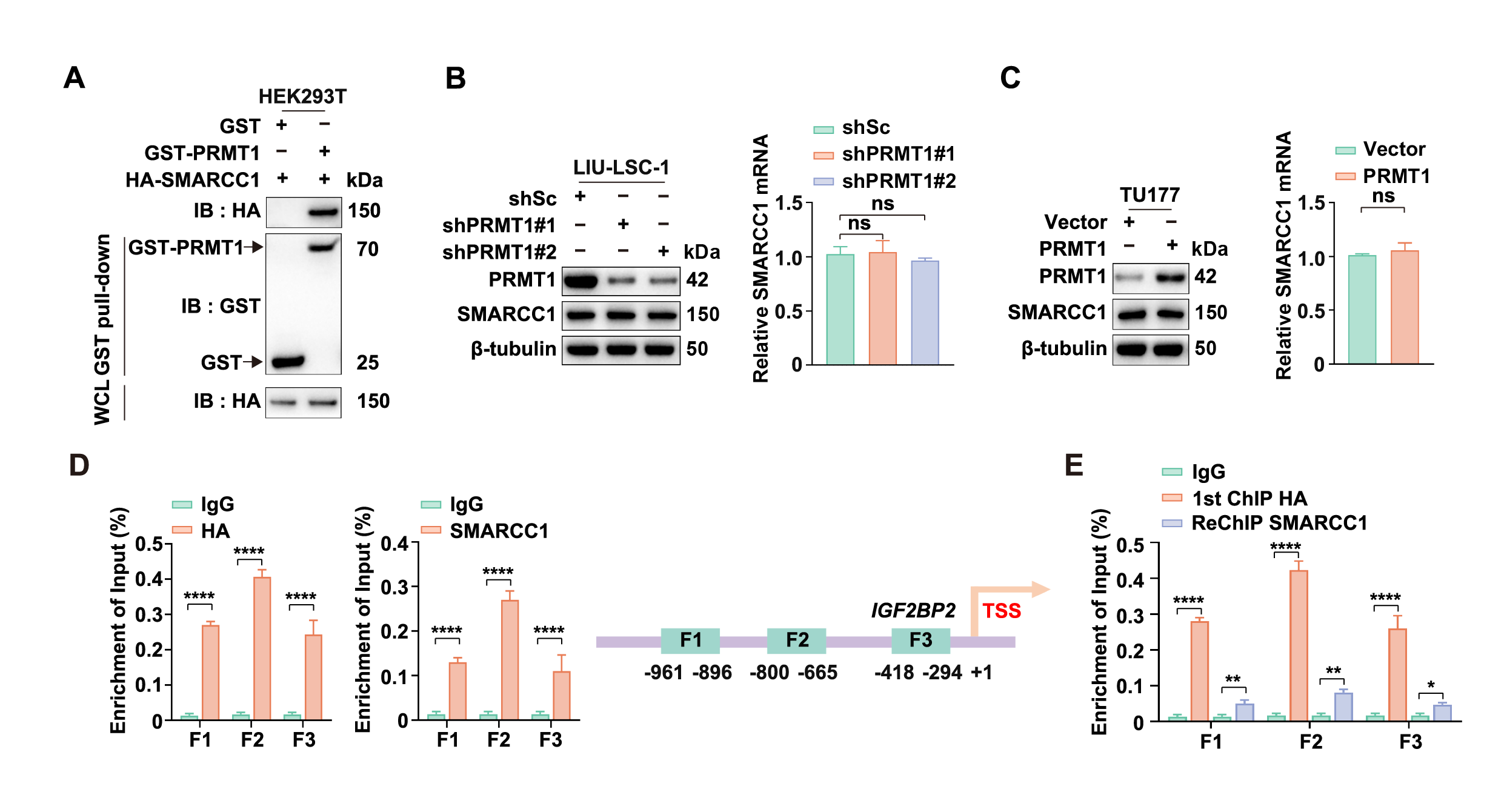


**Figure S9 PRMT1 directly binds to SMARCC1, and the interacting complex co-localizes in the promoter region of the *IGF2BP2* gene.**

A) Recombinant GST-PRMT1 was incubated with HEK293T cell lysates with ectopically expressing of HA-SMARCC1, followed by GST pull-down and immunoblotting analysis with GST and HA antibodies. B, C) LIU-LSC-1 cells were transfected with lentiviral shRNAs targeting PRMT1 (shPRMT1#1, shPRMT1#2) or a shSc (B). TU177 cells were transduced with either a control vector or lentiviruses expressing PRMT1 (C). The expression levels of SMARCC1 were assessed using western blotting (left panels) and qRT-PCR (right panels). Error bars represent mean ± SD. n.s.: not significant. D) ChIP analysis was conducted on HA-PRMT1-overexpressing TU177 cells, using antibodies against HA, SMARCC1, or control rabbit IgG. qPCR was performed to amplify the promoter region (F1, F2, and F3) of the *IGF2BP2* gene. E) qPCR quantification of DNA fragments from the *IGF2BP2* promoter region (F1, F2, and F3) recovered after ChIP using control rabbit IgG or anti-HA antibody, followed by reChIP using anti-SMARCC1 antibody or control rabbit IgG in TU177 cells overexpressing HA-PRMT1. Error bars represent mean ± SD. n.s.: not significant; ****P < 0.0001.


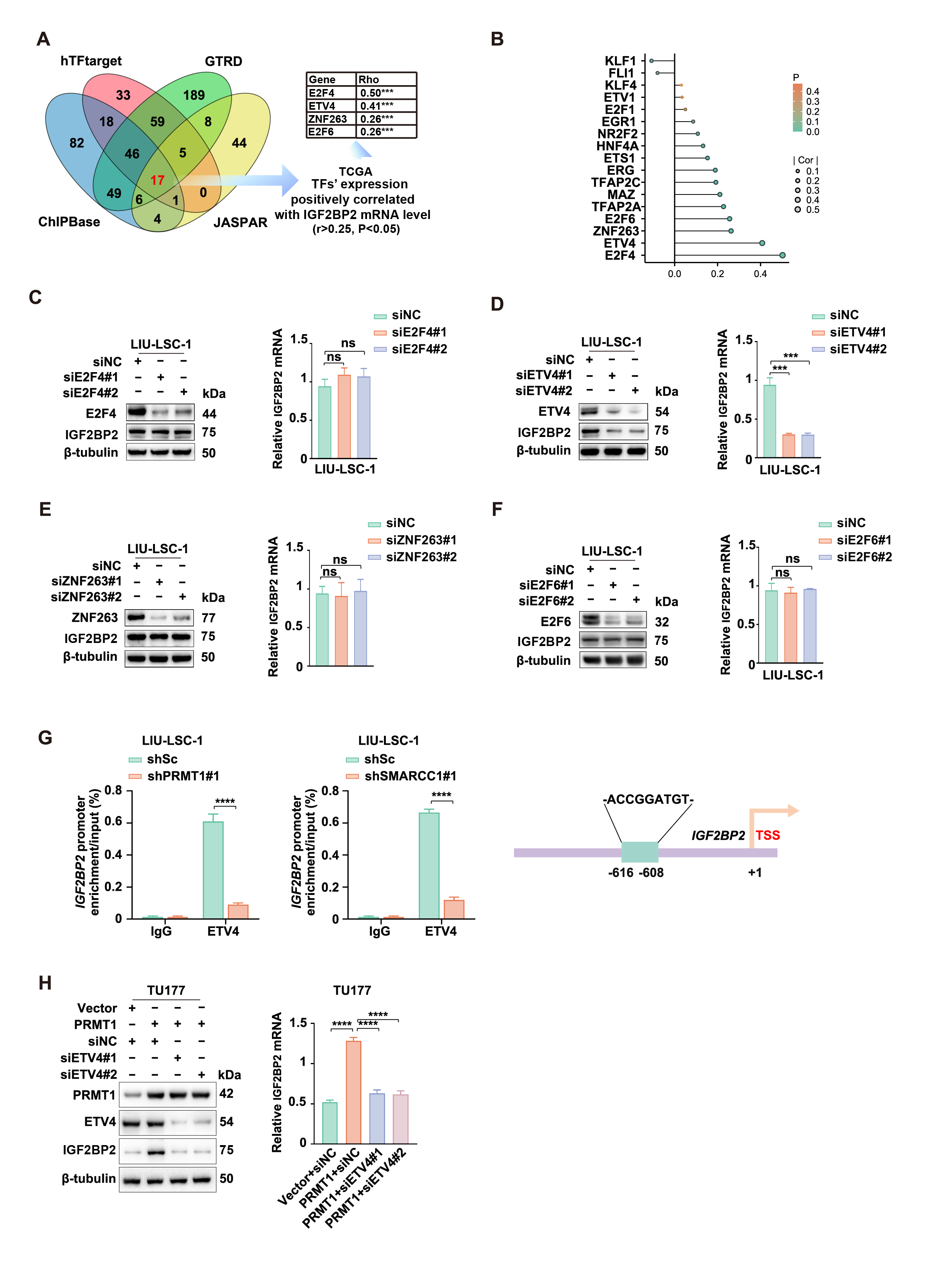


**Figure S10 ETV4 promotes the transcriptional activation of *IGF2BP2*.**

A, B) Flowchart for screening four potential TFs of IGF2BP2. The Venn diagram displays 17 TFs identified from four databases (A). A correlation analysis of these 17 TFs with the mRNA levels of IGF2BP2 was performed using the TCGA-HNSCC dataset (R > 0.25, p < 0.05) showed by lollipop Plot (B). C-F) LIU-LSC-1 cells were transfected with siRNAs targeting E2F4, ETV4, ZNF263 and E2F6, or with siNC for 48 h. G) ChIP analysis was conducted on shPRMT1#1, shSMARCC1#1 or shSC LIU-LSC-1 cells, using antibodies against ETV4, or control rabbit IgG. qPCR was performed to amplify the promoter region of the *IGF2BP2* gene. H) siETV4#1, siETV4#2, or siNC were transfected into PRMT1-overexpressing TU177 cells for 48 h. C-F, H) The expression levels of IGF2BP2 were assessed using western blotting (left panels) and qRT-PCR (right panels). Error bars represent mean ± SD. **P < 0.01, ***P < 0.001, ****P < 0.0001. n.s.: not significant.


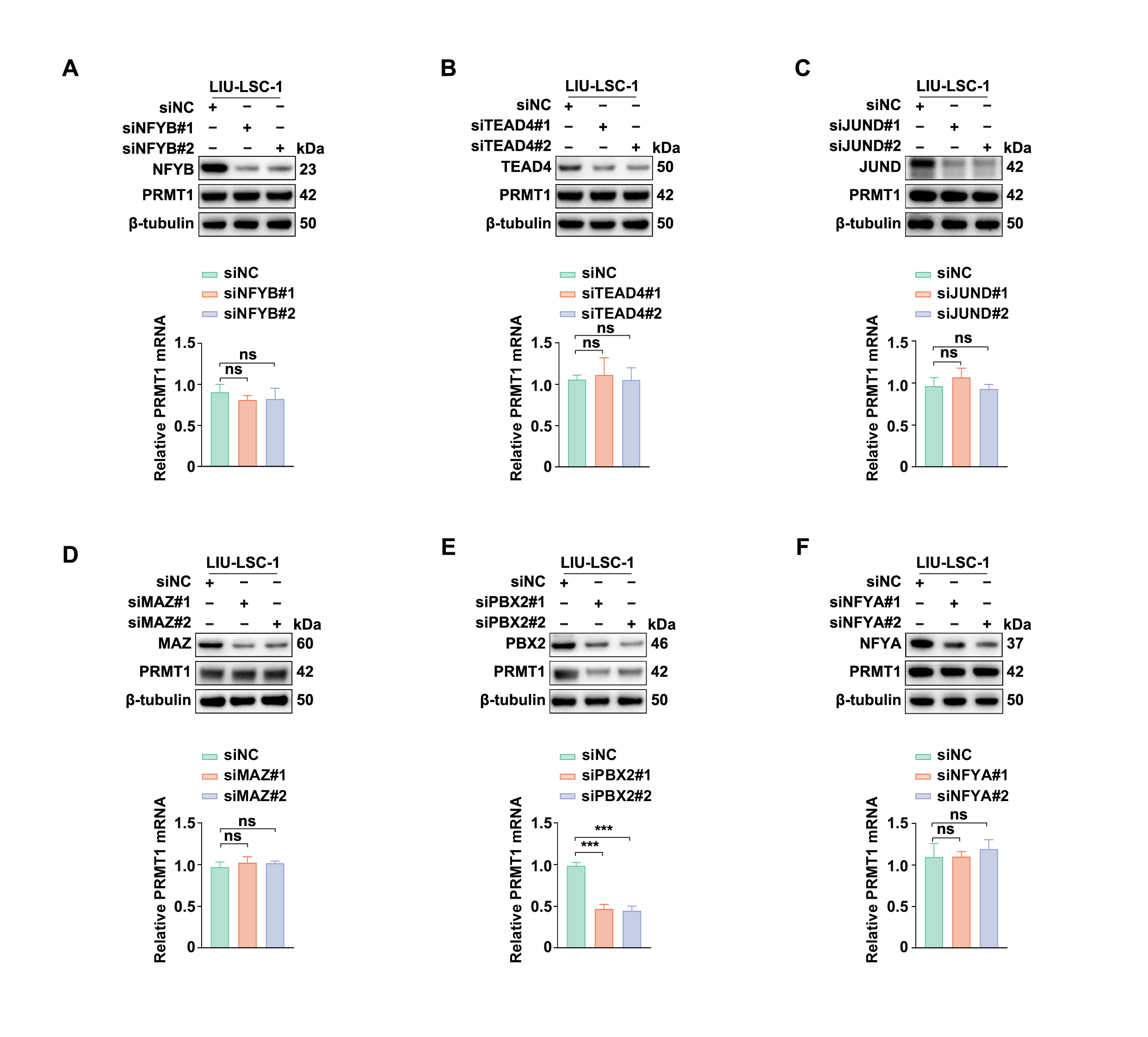


**Figure S11 Knockdown of PBX2, rather than the other five transcription factors (NFYB, TEAD4, JUND, MAZ, and NFYA), downregulates the expression of PRMT1.**

LIU-LSC-1 cells were transfected with siRNAs targeting NFYB, TEAD4, JUND, MAZ, PBX2, and NFYA, or with siNC for 48 h. The expression levels of PRMT1 were assessed using western blotting (upper panels) and qRT-PCR (lower panels). Error bars represent mean ± SD. n.s.: not significant; ***P < 0.001.


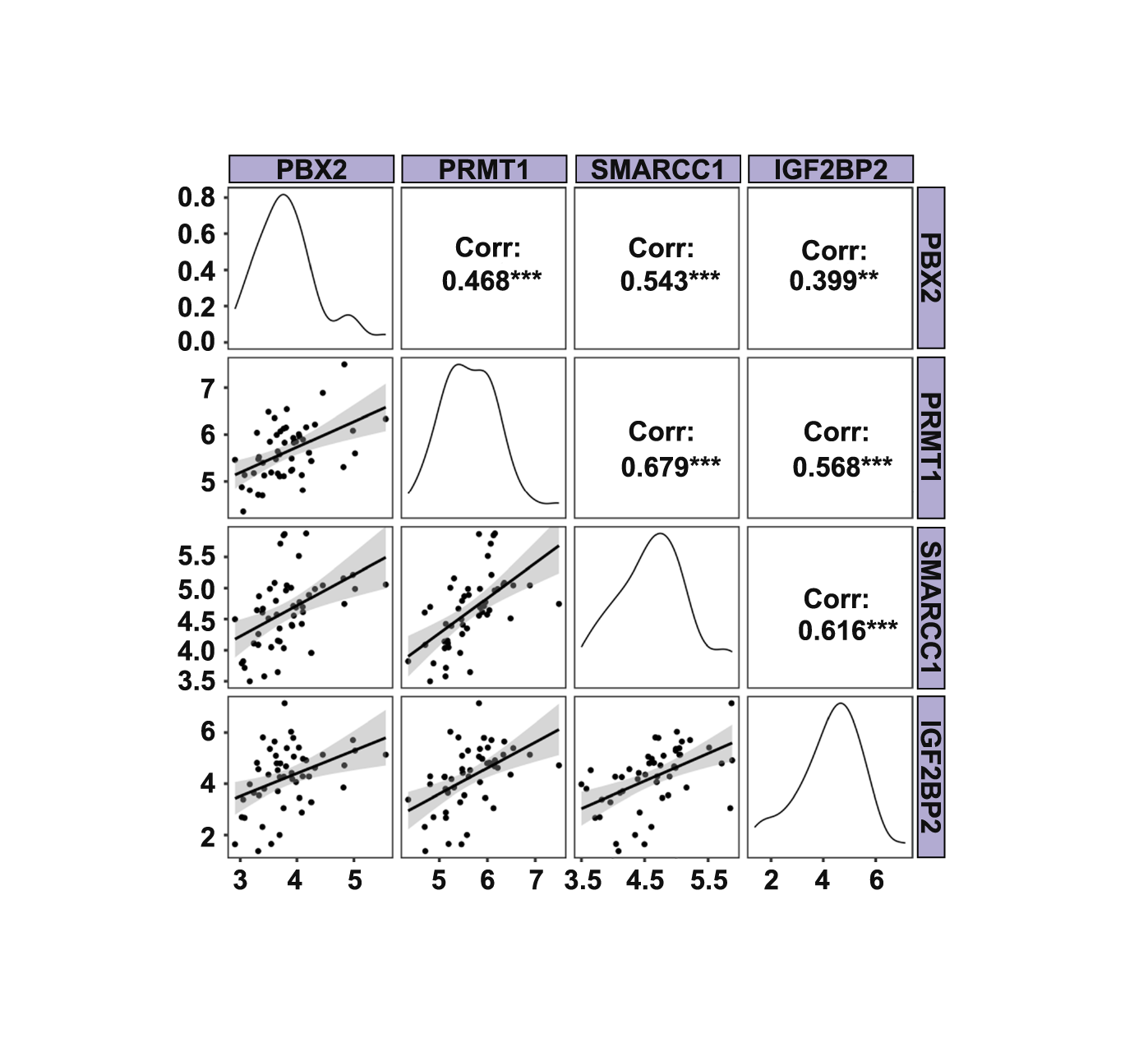


**Figure S12 A positive correlation exists among the expression levels of PBX2, PRMT1, SMARCC1, and IGF2BP2 in HNSCC tissues.**

The correlation of expression levels among PBX2, PRMT1, SMARCC1, and IGF2BP2 in HNSCC tissues from the GSE130605 dataset was analyzed. Spearman’s correlation coefficient values are indicated in the upper right corner. Scatterplot matrices with fitted trend lines for the respective genes are presented in the lower left corner. Error bars represent the mean ± SD. **P < 0.01, ***P < 0.001.


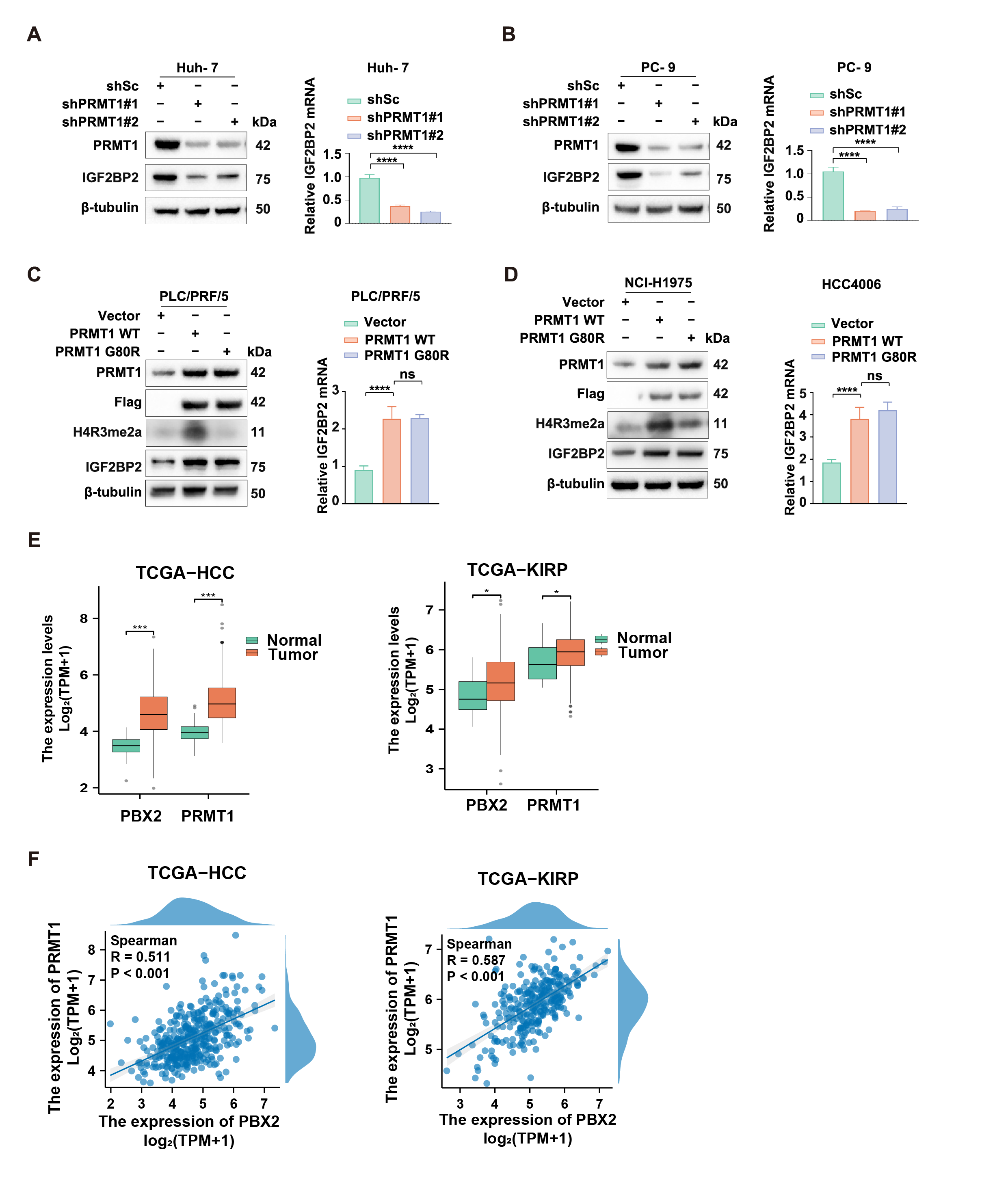


**Figure S13** **Discovery and implicate the PBX2–PRMT1–SWI/SNF–IGF2BP2 Axis in various cancers.**

A, B) Huh-7(A) and PC-9(B) cells were transfected with siRNAs targeting PRMT1, or with siNC for 48 h. C, D) PLC/PRF/5(C) and NCI-H1975 (D) cells were transfected with wild-type PRMT1 or PRMT1 G80R mutant constructs. The expression levels of IGF2BP2 were assessed using western blotting (left panels) and qRT-PCR (right panels). Error bars represent mean ± SD. n.s.: not significant; ****P < 0.0001. E) Analysis of PBX2 and PRMT1 expression in tumor and normal tissues using TCGA-HCC and TCGA-KIRP datasets. Error bars representing the mean ± SD. *P < 0.05, ***P < 0.001. F) The correlation between PBX2 and PRMT1 mRNA expression levels in TCGA-HCC and TCGA-KIRP tumor tissues was analyzed, and Pearson’s correlation coefficients were calculated.


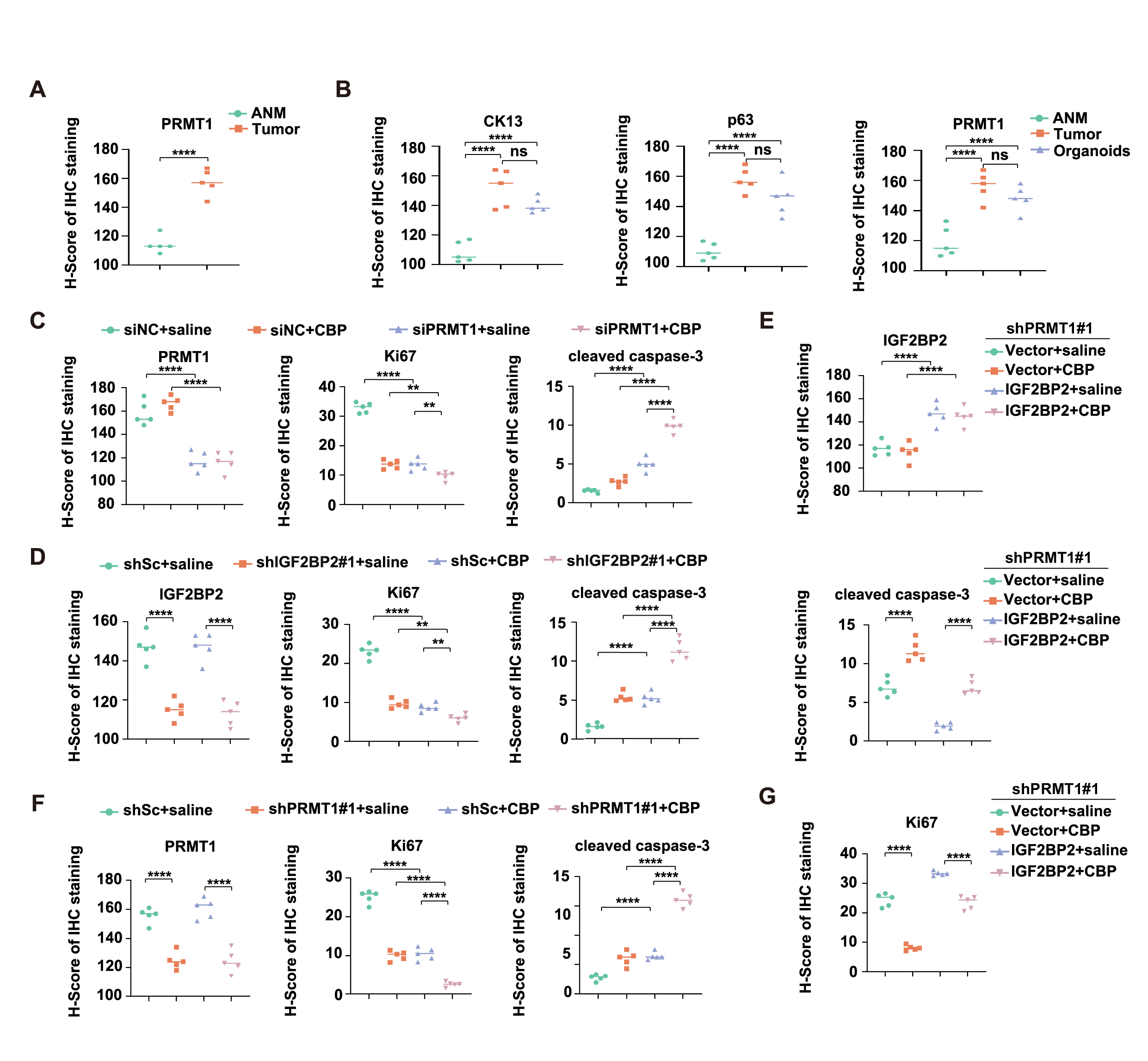


**Figure S14 The quantification of IHC staining.**

The IHC staining results in Figure 1F, Figure 1K, Figure 2G, Figure 3O, Figure 4N, Figure S4F, and Figure S7G were assessed using H-scores (p63, CK13, PRMT1, PBX2, SMARCC1 and IGF2BP2) or the percentage of positive area (Ki67 and cleaved caspase-3), and the quantitative analysis results are presented in A, B, C, D, E, F, and G respectively. Error bars indicate mean ± SD. *P<0.05; **P<0.01; ***P<0.001; ****P<0.0001. n.s: no significance.

**SUPPLEMENTARY TABLES**

**Supplementary Table S8.** Clinical features of 85 HNSCC patients

| Characteristics | Number of Cases (%) |
| --- | --- |
| Age |  |
| < 60 | 34(40) |
| ≥ 60 | 51(60) |
| Gender |  |
| Female | 8(9.4) |
| Male | 77(90.6) |
| T Stage |  |
| T1 | 14(16.5) |
| T2 | 21(24.7) |
| T3 | 23(27.1) |
| T4 | 27(31.8) |
| lymph node metastasis |  |
| N0 | 59(69.4) |
| N+ | 26(30.6) |
| Distant metastasis |  |
| M0 | 83(97.6) |
| M1 | 2(2.4) |
| Tumor location |  |
| Hypopharynx | 33(38.8) |
| Larynx | 52(61.2) |

**Supplementary Table S9.** Clinical features of 12 HNSCC patients

| Characteristics | Number of Cases (%) |
| --- | --- |
| Age |  |
| < 60 | 4(33.3) |
| ≥ 60 | 8(66.7) |
| Gender |  |
| Female | 2(16.7) |
| Male | 10(83.3) |
| T Stage |  |
| T1 | 2(16.7) |
| T2 | 3(25) |
| T3 | 4(33.3) |
| T4 | 3(25) |
| lymph node metastasis |  |
| N0 | 7(58.3) |
| N+ | 5(41.7) |
| Distant metastasis |  |
| M0 | 12(100) |
| M1 | 0(0) |
| Tumor location |  |
| Hypopharynx | 7(58.3) |
| Larynx | 5(41.7) |

**Supplementary Table S10.** Clinical characteristics of donor patients

| Clinical characteristics of PDO donor patients | | | | | | |  |
| --- | --- | --- | --- | --- | --- | --- | --- |
| NO. | Age | Gender | T stage | Cancer lymph node metastasis | Distant metastasis | Cancer type | HPV  Status |
| Patient 1 | 59 | Male | T3 | N1 | M0 | Hypopharyngeal squamous cell carcinoma | negative |

| Clinical characteristics of PDX donor patient | | | | | | |  |
| --- | --- | --- | --- | --- | --- | --- | --- |
| NO. | Age | Gender | T stage | Cancer lymph node metastasis | Distant metastasis | Cancer type | HPV  Status |
| Patient 1 | 50 | Male | T3 | N1 | M0 | Laryngeal squamous cell carcinoma | negative |

**Supplementary Table S11.** Cell lines and growth medium

| Cell line | Source | Tissue source | Complete growth medium |
| --- | --- | --- | --- |
| TU177 | Otwo Biotech Inc. (Shenzhen, China). | HNSCC | RPMI 1640 (Gibco: Cat#11875093) + 10% FBS (Gibco: Cat#10099141C) + 1% penicillin/streptomycin (Beyotime, Cat# C0222) |
| LIU-LSC-1 | newly established cell line | HNSCC | Epithelial Cell Complete Medium (VivaCell: Cat#C3650-0100) + 1% penicillin/streptomycin (Beyotime, Cat# C0222) |
| NOK | Otwo Biotech Inc. (Shenzhen, China). | Normal oral mucosal tissues | RPMI 1640 (Gibco: Cat#11875093) + 10% FBS (Gibco: Cat#10099141C) + 1% penicillin/streptomycin (Beyotime, Cat# C0222) |
| FaDu | ATCC (Manassas, VA, USA) | HNSCC | RPMI 1640 (Gibco: Cat#11875093) + 10% FBS (Gibco: Cat#10099141C) + 1% penicillin/streptomycin (Beyotime, Cat# C0222) |
| TU686 | BeNa Culture Collection (Beijing, China) | HNSCC | RPMI 1640 (Gibco: Cat#11875093) + 10% FBS (Gibco: Cat#10099141C) + 1% penicillin/streptomycin (Beyotime, Cat# C0222) |
| TU212 | Otwo Biotech Inc. (Shenzhen, China) | HNSCC | RPMI 1640 (Gibco: Cat#11875093) + 10% FBS (Gibco: Cat#10099141C) + 1% penicillin/streptomycin (Beyotime, Cat# C0222) |
| HEK-293T | ATCC (Manassas, VA, USA) | kidney; Embryo | DMEM (Gibco: Cat#11995065) + 10% FBS (Gibco: Cat#10099141C) + 1% penicillin/streptomycin (Beyotime, Cat# C0222) |
| Huh-7 | Otwo Biotech Inc. (Shenzhen, China) | HCC | DMEM (Gibco: Cat#11995065) + 10% FBS (Gibco: Cat#10099141C) + 1% penicillin/streptomycin (Beyotime, Cat# C0222) |
| PC-9 | Otwo Biotech Inc. (Shenzhen, China) | LUAD | RPMI 1640 (Gibco: Cat#11875093) + 10% FBS (Gibco: Cat#10099141C) + 1% penicillin/streptomycin (Beyotime, Cat# C0222) |
| PLC/PRF/5 | ATCC (Manassas, VA, USA) | HCC | DMEM (Gibco: Cat#11995065) + 10% FBS (Gibco: Cat#10099141C) + 1% penicillin/streptomycin (Beyotime, Cat# C0222) |
| NCI-H1975 | ATCC (Manassas, VA, USA) | LUAD | RPMI 1640 (Gibco: Cat#11875093) + 10% FBS (Gibco: Cat#10099141C) + 1% penicillin/streptomycin (Beyotime, Cat# C0222) |

**Supplementary Table S12.** Antibodies used in this study

| Antibodies | Source | Application |
| --- | --- | --- |
| Mouse monoclonal antibody anti-Ki-67 | Cell Signaling Technology (Cat# 9449) | 1:800 for IHC |
| Rabbit polyclonal antibody anti-β-Tubulin | Affinity Biosciences (Cat# AF7011) | 1:1000 for WB |
| Rabbit monoclonal antibody anti-PRMT1 | Abcam (Cat# ab190892) | 1:500 for IHC; 1:1000 for WB; 1:80 for IP; 1:2000 for IF |
| Rabbit polyclonal antibody anti-PRMT2 | Abcam (Cat# ab154154) | 1:1000 for WB |
| Rabbit monoclonal antibody anti-PRMT3 | Abcam (Cat# ab191562) | 1:10000 for WB |
| Rabbit monoclonal antibody anti-PRMT4 | Abcam (Cat# ab307091) | 1:1000 for WB |
| Rabbit monoclonal antibody anti-PRMT5 | Abcam (Cat# ab109451) | 1:10000 for WB |
| Rabbit monoclonal antibody anti-PRMT6 | Abcam (Cat# ab271091) | 1:1000 for WB |
| Rabbit monoclonal antibody anti-PRMT7 | Abcam (Cat# ab181214) | 1:1000 for WB |
| Rabbit polyclonal antibody anti-PRMT8 | Thermo Fisher Scientific (Cat# PA5-120639) | 1:1000 for WB |
| Rabbit polyclonal antibody anti-PRMT9 | Thermo Fisher Scientific (Cat# PA5-48942) | 1:1000 for WB |
| Rabbit monoclonal antibody anti-IGF2BP2 | Abcam (Cat# ab124930) | 1:200 for IHC; 1:2000 for WB; 1:100 for IF |
| Rabbit monoclonal antibody anti-Beta Actin | Proteintech (Cat# 66009-1-Ig) | 1:2000 for WB |
| Rabbit monoclonal antibody anti-CDH2 | Proteintech (Cat# 22018-1-AP) | 1:2000 for WB |
| Rabbit monoclonal antibody anti-MAD1L1 | Proteintech (Cat# 18322-1-AP) | 1:2000 for WB |
| Rabbit monoclonal antibody anti-SMARCC1 | Abcam (Cat# ab305037) | 1:100 for IHC; 1:1000 for WB; 1:30 for IP; 1:100 for IF |
| Rabbit monoclonal antibody anti-SMARCC1 | Cell Signaling Technology (Cat# 11956) | 1:100 for ChIP |
| Rabbit monoclonal antibody anti-SMARCA4 | Abcam (Cat# ab110641) | 1:10000 for WB |
| Rabbit monoclonal antibody anti-BRD7 | Cell Signaling Technology (Cat# 15125) | 1:1000 for WB |
| Mouse monoclonal antibody anti-SMARCD3 | Santa Cruz (Cat# sc-101163) | 1:200 for WB |
| Rabbit monoclonal antibody anti-SMARCE1 | Cell Signaling Technology (Cat# 33360) | 1:1000 for WB |
| Rabbit monoclonal antibody anti-E2F4 | Proteintech (Cat# 10923-1-AP) | 1:500 for WB |
| Mouse monoclonal antibody anti-ETV4 | Cell Signaling Technology (Cat# 65763) | 1:1000 for WB  1:200 for ChIP |
| Rabbit monoclonal antibody anti-ZNF263 | Zenbio (Cat# 124013) | 1:1000 for WB |
| Rabbit monoclonal antibody anti-E2F6 | Proteintech (Cat# 10691-1-AP) | 1:500 for WB |
| Rabbit polyclonal antibody anti-PBX2 | Abcam (Cat# ab252996) | 1:200 for IHC; 1:500 for WB |
| Rabbit polyclonal antibody anti-NFYB | Abcam (Cat# ab111577) | 1:500 for WB |
| Rabbit monoclonal antibody anti-TEAD4 | Abcam (Cat# ab308621) | 1:1000 for WB |
| Rabbit monoclonal antibody anti-JUND | Abcam (Cat# ab181615) | 1:1000 for WB |
| Rabbit polyclonal antibody anti-MAZ | Abcam (Cat# ab85725) | 1:2000 for WB |
| Rabbit monoclonal antibody anti-NFYA | Abcam (Cat# ab139402) | 1:1000 for WB |
| Rabbit polyclonal antibody anti-H4R3me2a | Thermo Fisher Scientific (Cat# PA5-102612) | 1:500 for WB |
| Goat Anti-Mouse IgG HRP | Abcam (Cat# ab6789) | 1:2000 for WB |
| Goat Anti-Rabbit IgG HRP | Abcam (Cat# ab6721) | 1:2000 for WB |
| Mouse monoclonal anti-Rabbit IgG light chain | Abcam (Cat# 99696) | 1:2000 for WB |
| Rabbit monoclonal antibody anti-GAPDH | Cell Signaling Technology (Cat #2118) | 1:1000 for WB |
| Rabbit polyclonal antibody anti-p63 | Proteintech (Cat # 12143-1-AP) | 1:200 for IHC |
| Mouse monoclonal antibody anti-Cytokeratin 13 | Proteintech (Cat #66684-1-lg) | 1:1000 for IHC |
| Rabbit polyclonal antibody anti-Cleaved Caspase-3 | Cell Signaling Technology (Cat# 9661) | 1:400 for IHC; 1:400 for IF |
| Mouse monoclonal antibody anti-Flag | Cell Signaling Technology (Cat #8146) | 1:1000 for WB; 1:50 for IP |
| Rabbit monoclonal antibody anti-HA | Cell Signaling Technology (Cat #3724) | 1:1000 for WB; 1:50 for IP; 1:50 for ChIP |

**Supplementary Table S13.** Targeting sequences for siRNAs used in this study

| **Note** | **Sequences (5'-3')** |
| --- | --- |
| siNC | UUCUCCGAACGUGUCACGU |
| siPRMT1#1 | GCUACUGCCUCUUCUACGA |
| siPRMT1#2 | AUACUGGAACACUCGAUCC |
| siPRMT2#1 | GCUGAUAAGGAUUAUCGUA |
| siPRMT2#2 | GUCUCUUCUGUGCACACUA |
| siPRMT3#1 | CCGAGAUUUCAUAUACCAA |
| siPRMT3#2 | GCAUGAAGAAAGCAGUUAU |
| siPRMT4#1 | GUCUGCUUAUUGCCAACAA |
| siPRMT4#2 | GCUACAUGCUCUUCAACGA |
| siPRMT5#1 | GCUAGACCGAGUACCAGAA |
| siPRMT5#2 | GGAUUCGUCCAGACUCAAA |
| siPRMT6#1 | GGCAUUCUGAGCAUCUUCU |
| siPRMT6#2 | ACCAGUGGAGACUGUAGAG |
| siPRMT7#1 | CUCGGUUUGGAGAGAUCAA |
| siPRMT7#2 | UCUGUCUUUGUCAUGUAGC |
| siPRMT8#1 | GGAACUCCAUGUACCACAA |
| siPRMT8#2 | GGUAGCGAUUGAAGACAGA |
| siPRMT9#1 | CGAAGUCACUUGACAUAGA |
| siPRMT9#2 | GGAACUGGACAGAGUAAUA |
| siNFYA#1 | GAGCUAAACUAGAGGCAGA |
| siNFYA#2 | GGACAAGGUCAAACCAUCA |
| siTEAD4#1 | GACAGAGUAUGCUCGCUAU |
| siTEAD4#2 | GCAGUCAGGCACUGGACAA |
| siSP2#1 | GGUCACGAAGAACUUGUAA |
| siSP2#2 | GGUGUUCGCUAUCCAGAAU |
| siMAZ#1 | GCCUUGGAGAAGAAGACAA |
| siMAZ#2 | GUCAGACAAGUGCACUCAA |
| siNFYB#1 | CAUUGCGUGUACAGACCUU |
| siNFYB#2 | GUAAGCGGCUGGGUUAAUU |
| siPBX2#1 | GAAUCACUCCGACACUCGA |
| siPBX2#2 | GCAUGUAAUGAGUUCACGA |
| siSMARCC1 | CCCACCACAUUUACCCAUA |
| siSMARCA4 | GGUGGACUACAGCGACUCA |
| siBRD7 | CCGCAUUAUGACUCCACAU |
| siSMARCD3 | GACGAUUGAGUCCAUAAAC |
| siSMARCE1 | CCGCGUACCUUGCUUACAU |
| siCDH2#1 | GGACCGAGAAUCACCAAAU |
| siCDH2#2 | GACCAUCACUCGGCUUAAU |
| siMAD1L1#1 | GGCAGUGUCAGCAGAACUU |
| siMAD1L1#2 | GGCUACCAGAUCGACAUCA |
| siIGF2BP2#1 | CCUAGCUGUUUAUCGACGGAA |
| siIGF2BP2#2 | GCCGUUGUCAACGUCACAUAU |
| siZNF263#1 | AAUCAGCUGAAGGUCAAC |
| siZNF263#2 | CUCUCAUAGAUCCAAUUU |
| siE2F4#1 | CGGATTTACGACATTACCAAT |
| siE2F4#2 | GACCTCTTTGATGTGCCTGTT |
| siE2F6#1 | CGATGTCTATTTGTGTGAAGT |
| siE2F6#2 | GAAATCCAAGAACCATATTAG |
| siETV4#1 | CAGTGCCTTTACTCCAGTGCC |
| siETv4#2 | CTCAGGAAATTCCGTTGCTCT |

**Supplementary Table S14.** Primer sequences for qRT-PCR used in this study

| Prime name | Primer sequence (5'-3') |
| --- | --- |
| (human) PRMT1 FORWARD | TTCACACGCTGCCACAAGAGG |
| (human) PRMT1 REVERSE | ATGCCGATGGTGCCGAAGATC |
| (human) β-actin FORWARD | CCTGGCACCCAGCACAAT |
| (human) β-actin REVERSE | GGGCCGGACTCGTCATAC |
| (human) PBX2 FORWARD | CAGGCCACTGAGGTCCTAAA |
| (human) PBX2 REVERSE | TGTTGCCAAACCAGTTGGAG |
| (human) PRMT2 FORWARD | CCCAGAAGTGAATCGCAGGG |
| (human) PRMT2 REVERSE | TGCAGTGGTTTGTCTCAGGATA |
| (human) PRMT3 FORWARD | GTACCCTTCTCATACCCCAATGG |
| (human) PRMT3 REVERSE | GACGAGCAGGTTCTGACATCT |
| (human) PRMT4 FORWARD | TCGCCACACCCAACGATTT |
| (human) PRMT4 REVERSE | GTACTGCACGGCAGAAGACT |
| (human) PRMT5 FORWARD | CTGTCTTCCATCCGCGTTTCA |
| (human) PRMT5 REVERSE | GCAGTAGGTCTGATCGTGTCTG |
| (human) PRMT6 FORWARD | TACCGCCTGGGTATCCTTCG |
| (human) PRMT6 REVERSE | CCTGTTCCGGCAACTCTACA |
| (human) PRMT7 FORWARD | TTGACACAGAGCTGATCGGG |
| (human) PRMT7 REVERSE | CAACGGGAGGGACGATGAC |
| (human) PRMT8 FORWARD | CCTGCTAAGCCCGTGCAAT |
| (human) PRMT8 REVERSE | TGGGCATAGGAGTCGAAGTAA |
| (human) PRMT9 FORWARD | AGGACTTCGGCACTGCCTAT |
| (human) PRMT9 REVERSE | TCCTTCACGTCGTGTTTCAGC |
| (human) IGF2BP2 FORWARD | AGTGGAATTGCATGGGAAAATCA |
| (human) IGF2BP2 REVERSE | CAACGGCGGTTTCTGTGTC |
| (human) SMARCC1 FORWARD | AGCTGTTTATCGACGGAAGGA |
| (human) SMARCC1 REVERSE | GCATCCGCATGAACATACTTCTT |
| (human) NFYA FORWARD | CAGTGGAGGCCAGCTAATCAC |
| (human) NFYA REVERSE | CCAGGTGGGACCAACTGTATT |
| (human) TEAD4 FORWARD | GAACGGGGACCCTCCAATG |
| (human) TEAD4 REVERSE | GCGAGCATACTCTGTCTCAAC |
| (human) SP2 FORWARD | CTCAGCCCCGGCAAGAATAG |
| (human) SP2 REVERSE | TTGATCGGGTCCCTTTGTTGA |
| (human) MAZ FORWARD | ACCACCTGAACCGACACAAG |
| (human) MAZ REVERSE | AAGCTGCCTCACATTTCTCAC |
| (human) NFYB FORWARD | ATGACAATGGATGGTGACAGTTC |
| (human) NFYB REVERSE | CTAGCCACGTTTGCTATTGGA |
| (human) CDH2 FORWARD | CGATAAGGATCAACCCCATACA |
| (human) CDH2 REVERSE | TTCAAAGTCGATTGGTTTGACC |
| (human) MAD1L1 FORWARD | GAAGATGCTGAAGTCTCAGTCCA |
| (human) MAD1L1 REVERSE | CGACCTTCAACCTGAGCGTG |
|  |  |

**Supplementary Table S15.** Targeting sequences for shRNẠs used in this study

| **Note** | **Sequences (5'-3')** |
| --- | --- |
| shSc | TTCTCCGAACGTGTCACGT |
| shPRMT1#1 | GGCCTACTTCAACATCGAGTT |
| shPRMT1#2 | GGAAGCAGACGGTGTTCTACA |
| shIGF2BP2#1 | CCTAGCTGTTTATCGACGGAA |
| shIGF2BP2#2 | GCCGTTGTCAACGTCACATAT |
| shSMARCC1#1 | CCCACCACATTTACCCATATT |
| shSMARCC1#2 | GCTATGATACTTGGGTCCATA |
| shPBX2#1 | GAATCACTCCGACACTCGA |
| shPBX2#2 | GCATGTAATGAGTTCACGA |

**Supplementary Table S16.** Primers used for ChIP Assays in this study

|  | **Sense (5'-3')** | **Antisense (5'-3')** |
| --- | --- | --- |
| PBX2 binding site (PCR/ qPCR) | CGTGACGTCGCTTCCGGATAA | CCCTCCCCTTTACACCCAGCA |
| IGF2BP2 F1(qPCR) | TGGGCGGAGAGGACGTGACT | GGCCTCAGTTTCCCGACCG |
| IGF2BP2 F2(qPCR) | ACACGCGCCGCTCGGTCACC | AGGCGAGGGCAGGCTGGGAG |
| IGF2BP2 F3(qPCR) | ACCCGCTTCCGCCCGCCTGG | AGCCGAGAGGGAGCGAGCGAGC |
| ETV4 binding site (qPCR) | CTCCCAGCCTGCCCTCGCCTCT | TGAGCGCGGAGAGGGGTGTG |
